# Bayesian-knowledge driven ontologies: A framework for fusion of semantic knowledge under uncertainty and incompleteness

**DOI:** 10.1371/journal.pone.0296864

**Published:** 2024-03-27

**Authors:** Eugene Santos, Jacob Jurmain, Anthony Ragazzi

**Affiliations:** Thayer School of Engineering, Dartmouth College, Hanover, NH, United States of America; University of Pretoria, SOUTH AFRICA

## Abstract

The modeling of uncertain information is an open problem in ontology research and is a theoretical obstacle to creating a truly semantic web. Currently, ontologies often do not model uncertainty, so stochastic subject matter must either be normalized or rejected entirely. Because uncertainty is omnipresent in the real world, knowledge engineers are often faced with the dilemma of performing prohibitively labor-intensive research or running the risk of rejecting correct information and accepting incorrect information. It would be preferable if ontologies could explicitly model real-world uncertainty and incorporate it into reasoning. We present an ontology framework which is based on a seamless synthesis of description logic and probabilistic semantics. This synthesis is powered by a link between ontology assertions and random variables that allows for automated construction of a probability distribution suitable for inferencing. Furthermore, our approach defines how to represent stochastic, uncertain, or incomplete subject matter. Additionally, this paper describes how to fuse multiple conflicting ontologies into a single knowledge base that can be reasoned with using the methods of both description logic and probabilistic inferencing. This is accomplished by using probabilistic semantics to resolve conflicts between assertions, eliminating the need to delete potentially valid knowledge and perform consistency checks. In our framework, emergent inferences can be made from a fused ontology that were not present in any of the individual ontologies, producing novel insights in a given domain.

## 1 Introduction

Ontologies, the foundation of the semantic web, are widely used in machine knowledge representation. They are used to define classes and the relationships between their members within a domain. Reasoning algorithms reveal implicit knowledge in the model according to the rules of description logic (DL) [1] which is a decidable subset of predicate calculus. Unfortunately, DL does not conveniently represent uncertainty, the existence of multiple conflicting possible states of a domain. There are several approaches to introducing strong uncertainty semantics into DL. Two prominent approaches which have enjoyed some success are fuzzy logic and possibility theory. These have been applied in frameworks such as Fuzzy OWL [2] and possibilistic description logic [3]. However, in both theories, some interactions between variables are lost during inferencing. The lost information may be unnecessary for modeling the notions of fuzzy set membership and possibility, but are unable to capture a more complex notion of uncertainty which supports chains of “if-then” interactions between variables. One uncertainty theory which has strong semantics and fully captures these variable interactions is probability theory. Unfortunately, to the best of our knowledge, all the representation frameworks for ontologies which are rooted in probability theory exhibit lossy reasoning or have counterintuitive restrictions on their flexibility. The probabilistic DLs based on Nilsson’s probabilistic logic [4] experience decay in relative precision during reasoning due to their expression of probabilities as intervals. Approaches using Bayesian Networks (BNs) [5], such as BayesOWL [6], MEBN/PR-OWL [7], and P-CLASSIC [8], contain a representation granularity mismatch: Bayesian Networks require complete specification of the domain’s probability distribution with no incompleteness, but ontologies have a finer granularity which allows for incompleteness. Some domains with incompletely defined relationships can only be represented in Bayesian Network based frameworks by over defining them. We address all these issues in more detail in Section 2.

There exists another probabilistic knowledge representation framework that can be unified with description logic. Bayesian Knowledge Bases [9, 10], or BKBs, are designed to handle incompleteness, and they do not experience reasoning decay like other uncertainty logics. BKBs represent domain knowledge as sets of “if-then” conditional probability rules between propositional variable instantiations. They use those conditional probabilities to compute marginal probabilities of the domain’s instantiations, or states. BKBs represent knowledge with the same granularity as ontologies, but they are not an immediate substitute for them because they only reason about propositional knowledge, not predicated knowledge like ontologies do. A synthesis of BKBs and DL which preserves the capabilities of both is desirable. This paper presents an approach for representing uncertainty in ontologies with probability semantics as well as the ability to naturally fuse multiple dissonant probabilistic ontologies which otherwise could not be formally reconciled.

This paper presents two broad contributions. First, we extend a preliminary formulation of the knowledge representation and reasoning framework called Bayesian Knowledge-driven Ontologies (BKOs) [11]. BKOs unite the predicate reasoning capabilities of DL with the probabilistic reasoning capabilities of BKBs. They represent knowledge as predicate logic assertions like DL, but also represent conditional probability rules between those assertions like BKBs. We will show that a BKO can reason about both types of knowledge without disempowering either, based on four points:

- Uncertainty is defined as the presence of multiple possible states of the world where we have insufficient knowledge to determine which state is true, but such that we can define a probability distribution over the possible states.
- For any set of mutually disjoint classes in an ontology, any individual can be a member of at most one of those classes. Therefore, potential class assignments between the individual and the classes can be represented as assignments of a discrete random variable.
- Generalizing the rule of universal instantiation to its probabilistic analog allows uncertainty to be propagated from terminological axioms to the assertional axioms they imply.
- A BKO where all implicit knowledge has been made explicit maps to an equivalent BKB.

Second, this paper demonstrates that BKO theory allows for reasoning over multiple fused ontologies, including dissonant ones, without modifying them. This is an improvement over current methods of resolving conflicts in merged ontologies, which resort to modifying them up to the point of rejecting knowledge completely (see [12] for an example). Recent work [13] has pushed this envelope, introducing computational methods for minimizing the number of assertions deleted. We make the distinction between “merged” and “fused” ontologies. While both refer to combining multiple ontologies into one larger one, we describe “merged” ontologies as ones that require some manual or automated altering of information and “fused” ontologies as ones that do not require any alterations. Methods for ontology merging compromise a source’s potentially valid perspective and miss opportunities for fusion-derived insights. Our methods of fusing ontologies without altering them means BKO theory can take advantage of every potential insight it is provided with. Provided they are lexically aligned, independent machine reasoning can be performed on dissonant ontologies from diverse sources. Even the requirement for lexical alignment is soft—where the source ontologies are not lexically aligned, including one or more alignment ontologies as inputs to the fusion algorithm is sufficient to ensure a valid result. This could be done with manually curated bridge ontologies or by applying recent work on automated ontology alignment [14, 15]. In Section 7 we fuse two biological ontologies involving the sciatic nerve, the largest nerve in the body that has gained much attention in biomedical research. This example highlights some of the strengths of BKO fusion, specifically the ability to reason despite contradictions and how emergent information can be generated only through fusion.

Our paper is organized as follows: We begin in Section 2 with a brief survey of representative prior approaches to augmenting DL with uncertainty semantics. Next, Sections 3 and 4 provide background on DL and BKB theory. Sections 4 and 5 define BKOs’ method of knowledge representation and reasoning. Section 6 defines the method of aligning and merging ontologies from different, potentially conflicting, sources. Section 7 walks through a detailed example of BKO reasoning over two fused biomedical ontologies. Finally, in Section 8, we provide our concluding remarks and a look at future directions and potential applications.

## 2 Related work

We now examine the two major classes of uncertainty semantics and their application into ontologies.

### 2.1 Fuzzy logic and possibility theory

Straccia [16] introduces fuzzy logic to semantic networks, while recent work can be found in Jain et al [17]. Fuzzy logic is an uncertainty theory designed to represent the notion of ambiguity using partial set membership. Fuzzy logic’s axioms are identical to probability theory, except that fuzzy logic lacks the axiom that the union of all events sums to one. The absence of that axiom means that fuzzy logic’s reasoning is a coarser treatment of information interaction, using min and max functions in place of the arithmetic functions that probability theory would use. Consider the following example: (Notation: for an individual or class *a*, a class *C*, and *p* ∈ [0, 1], *a* ∈ *C* : *p* states that *a* has membership in *C* with degree *p*.) Given the assertions *a* ∈ *C* : 0.7, *a* ∈ *D* : 0.4, *C* ∈ *E* : 0.2, and *D* ∈ *E* : 0.6, what is the membership of *a* in *E*? In simple fuzzy set theory, this is *max*(*min*(0.7, 0.2), *min*(0.4, 0.6)) = 0.4. Note that changes in the degree of different assertions may not affect the final result. A change in the degree of membership of *D* ∈ *E* would only alter the result if it dropped below 0.4, and a change in the degree of membership of *a* in *C* would not alter the result at all. This can be counterintuitive when we consider modeling any notion of causality, since we typically think that a change in a root variable should affect the result. Fuzzy logic is therefore more suited to its intended purpose of comparing entity descriptions than it is to capturing variable interactions.

Possibility theory is introduced to ontologies in [3]. Possibility theory models the notion of uncertainty of events, but like fuzzy logic it does not fully capture causal interactions. Possibility theory models the uncertainty of a single event with two numbers from the range [0, 1]: the event’s possibility, which is the degree to which the event could be expected to happen, and the event’s necessity, which is the degree to which the event must happen. These numbers are related in that the necessity of an event is equal to one minus the possibility of the event’s complement. Despite possibility theory’s sophisticated uncertainty representation capability, its reasoning mechanism still does not intuitively capture causality. Consider the following example and note the parallels to the example we used for fuzzy logic: (Notation: for events *A* and *C*, and *p*, *q* ∈ [0, 1] where *p* > *q*, *C*|*A* : (*p*, *q*) states that the possibility of *C* given *A* is *p* and the necessity of *C* given *A* is *q*.) Given the assertions *C*|*A*:(0.7, 0.5), *D*|*A* : (0.4, 0.3), *E*|*C* : (0.2, 0.1), and *E*|*D* : (0.6, 0.55), what is the possibility and necessity of *E* given *A*? The answer is simply that the possibility is *max*(*min*(0.7, 0.2), *min*(0.4, 0.6)) = 0.4 and the necessity is *max*(*min*(0.5, 0.1), *min*(0.3, 0.55)) = 0.3. As we discussed for fuzzy logic, this is a coarse treatment of causality.

### 2.2 Probability theory

We assume that the reader is familiar with the formulation and reasoning mechanics of probability theory, such as the notions of sample spaces, probability distributions, and conditional probabilities. Compare BKO theory to four groups of frameworks with similar reasoning goals: those founded in Nilsson’s probabilistic logic [4], Bayesian Networks [5], probabilistic Horn abduction [18], and lifted probabilistic inference [19].

Regarding Nilsson’s probabilistic logic-based frameworks, such as Lukasiewicz [20] (and more recently [21]), Halpern [22], and descendant works such as SHIQp [23], Prob-ALC [24], and Prob-EL [25], we see the difficulty they encounter in the following example: Recall that assertions in probabilistic DL are made probabilistic not by assigning them a probability, but by declaring an interval in which that probability is said to be found. This interval-based definition causes erosion of relative precision with every calculation. Suppose we have two probabilistic axioms, “Tweety is-a Bird” with probability between 0.70 and 0.80 (relative precision 0.13), and “Birds can Fly” with probability between 0.90 and 0.99 (relative precision 0.10). We wish to find the marginal probability that “Tweety can Fly”. Since the probabilities are only known as intervals, we must multiply their bounds to get the extreme cases of the marginal probability. The lowest possible probability is 0.9 × 0.7 = 0.63 and the highest possible probability is 0.8 × 0.99 = 0.79, so the marginal probability on “Tweety can Fly” is within the interval [0.63, 0.79]. Notice that this interval has a relative precision of 0.23, wider than either of the relative precisions on the original axioms. The representation of probabilities as intervals is an artifact of probabilistic DL’s foundation in Nilsson’s probabilistic logic [4], which is subject to the same decay in precision.

Regarding BN-based approaches, such as PR-OWL [26], BEL [27], Prob-Ont [28], BayesOWL [6], ByNowLife [29], and P-CLASSIC [8], consider the notion of incompleteness in a domain. Incompleteness is when the domain’s probability distribution could match one of a number of possible probability mass functions. Recall that BNs assume completeness by assuming that all variables whose joint distributions are not completely known are independent. Ontologies do not share this completeness assumption, so there are incomplete domains which can be represented with conventional ontologies but cannot be expressed with BN-based frameworks unless unsupported and potentially inaccurate constraints are included. Furthermore, we find notions which can be represented in semantic networks that are counterintuitive when we try to express them in BNs even with complete information. For example, if we wanted to describe the probability distribution between the variable “airplane model” and a discretized “gas mileage” variable, it would not make sense to define probabilities for the gas mileage of an engineless glider model. Even the notion of context-specific independence [30] does not avoid this problem because it would still require the “gas mileage” variable to have some distribution given a “glider model” value, but any distribution, even independence, is counterintuitive. Disregarding uncertainty, a semantic network would have no trouble expressing this domain’s concepts, because it could simply omit the glider’s gas mileage property from any consideration. Some approaches, such as PR-OWL, resolve this by defining a third truth value of “absurd”, but permitting incompleteness averts the need to contend with trinary logic.

Probabilistic Horn abduction [18] is a powerful and expressive knowledge modeling and reasoning framework with many conceptual and mathematical similarities to BKO theory, but it is prevented from discovering unanticipated explanations of the world by its demand that all hypotheses be independent and explicitly defined. In BKO theory those are unnecessary constraints, and relaxing them permits combination of knowledge through fusion as we shall detail in Section 6.

Lifted probabilistic inference [19] warrants special mention because it employs a similar assertion structure to that of BKOs, namely the assertion of conditional rules containing simple first-order terms taking individuals as arguments. However, the meanings of these terms and relationships are implicit and subject to interpretation, rather than explicit and richly expressed as in DL. So they do not allow for the autonomous reasoning capability of DL-driven knowledge models. Additionally, lifted probabilistic inference uses BNs to express uncertainty, and so runs afoul of their completeness requirement. Finally, lifted probabilistic inference does not require that the conditions of contradictory rules be mutually exclusive. Knowledge of which rule overrides another is kept implicit, and reasoning requires additional specifications to resolve. BKOs resolve these occurrences explicitly within the knowledge base through fusion.

Three additional approaches also merit mention for their use of structures similar to the conditional probability rules employed by BKBs. Do-calculus [31] arrived at a system which closely resembles conditional probability rules, though its formulation relies on very different intuitions than that of BKO theory. Do-calculus does not address the problem of modeling terminological knowledge, but it does formalize the fusion of conditional probability rules gathered under different regimes of population makeup and sampling bias. This is a matter which BKO theory delegates to the user, rolled up within the task of choosing source reliabilities. Our future work will seek to elaborate on our method of fusion to incorporate do-calculus’s insights and potentially subsume it. More recently, BLOG [32] also arrived at a knowledge representation system of conditional probability rules between logical assertions similar to that used by BKOs. However, BLOG does not aim to address the fusion of multiple probabilistic ontologies. We believe that BKOs subsume BLOG and that our fusion approach is directly applicable to multiple BLOGs which we intend to also explore in future work. Similarly, work by Jung and Lutz [33] is based on a definition of a probability distribution over possible states of the world akin to ours, but only defines assertional probabilistic rules, not terminological ones, and does not address fusion.

## 3 Background

Here we present necessary background information for the remainder of the paper. We first discuss DL with a focus on the ideas of consistency, assertional knowledge, and terminological knowledge. This is followed by a brief introduction to BKBs that includes their essential definitions and theorems. Finally we discuss BKB fusion, which as we will see has close ties to BKO fusion.

### 3.1 Description logic

We will briefly introduce a simple DL with definitions and notation based on set theory. These definitions are conceptually equivalent to formal DL as presented by Baader et al. [1], but are more closely related to set theory to simplify our derivations in the following sections. We ignore the possibility of mapping ontologies to multiple interpretations, and instead just consider classes and individuals as sets under a single interpretation. Multiple interpretations could be emulated using explicit namespace prefixes on concepts, individuals, etc.

The fundamental concept of description logic is the *class*, or *concept*, which is a set. An individual is an element of a class. A *role* is a binary operator acting from one individual (the owner) to another individual (the filler). Classes, individuals, and roles generally have real world interpretations, such as categories, objects, and relationships between objects.

While the words “class” and “concept” are for the most part interchangeable in DL, “class” generally refers to a more set-theoretic notion of classes/concepts as groups of individuals, while “concept” is used in the context of the descriptive nature of classes/concepts, i.e., that they characterize the nature of the individuals in them. We will mostly use “class” to emphasize the set-theoretic foundation of our theory.

*Atomic classes* are irreducible. They may be used in expressions called *constructors* to define new classes, called *constructed classes*. The expressiveness of constructors is specific to the DL being used. Simple construction operators are: complement, union, intersection, role existential quantification, and role value restriction. Additional operators are defined in more expressive DLs. In general, the more expressive a DL is, the longer its reasoning takes and the greater the risk of it being able to express undecidable problems. Ensuring decidability while achieving maximum expressivity is a hard problem in DL research.

Description logic makes the *open world assumption*: that the absence of a particular statement within a description of a domain does not imply that statement’s falsehood. This implies that every description is incomplete because we can always add new individuals, classes, and rules to it. Here lies an important and subtle distinction: the open world assumption does not imply that every domain is *necessarily* infinite, but does imply that every domain is *possibly* infinite, i.e. cannot be proven finite. For practical purposes we will assume than any description of a domain is finite, but we admit the possibility that the domain which it describes is infinite.

**Notation**. Denote the universal class, the class that contains all individuals, as ⊤ (down tack character, not the letter). Because ⊤ contains all individuals, it also contains all nonempty classes. ⊥ is the empty class, or the class that contains no individuals.

**Notation**. The complement of class *C* is written as ¬*C*, where ¬*C* = ⊤ − *C*

### 3.2 Asserting knowledge

In DL, knowledge is expressed through assertional axioms and terminological axioms. Assertional axioms are propositional: they characterize a single individual’s membership in classes. Terminological axioms are predicated: they define general rules applying to all individuals in a class. The set of assertional and terminological axioms in an ontology are often referred to as the A-box and the T-box, respectively.

**Definition 3.2.1**. An *assertional axiom* can be either a class assertion or a role assertion:

- A *class assertion* declares that *a* ∈ *C* for a class expression *C* and an individual *a*. DL commonly uses the notation *C*(*a*).
- A *role assertion* that *bRc* for a role expression *R* and individuals *b* and *c*. *bRc* states that c is a filler of the role *R* for an owner b. DL commonly uses the notations *R*(*b*, *c*) or (*b*, *c*):*R*.

**Definition 3.2.2**. A *terminological axiom* is a statement asserting a relation between two classes.

Some standard forms of terminological axioms in DL are subsumption, equivalence, and disjointness axioms. For classes *C* and *D*,

- A subsumption axiom is of the form *C* ⊆ *D*
- An equivalence axiom is of the form *C* = *D*
- A disjointness axiom is of the form *C* ∩ *D* = ⊥

In some ontology languages, such as the variants of OWL, knowledge can be presented and used in the form of property characteristics [34], which define specific inference rules for instantiations of properties such as functionality, transitivity, and symmetry. This expressive capability is often useful, but somewhat ad-hoc. In this paper we only consider formal, decidable DLs, and therefore only use property characteristics that can be directly expressed in them.

The notion of consistency between assertions is an important one in DL. While typically used for error-checking after reasoning, we will rely on it heavily in defining probabilistic relationships.

**Definition 3.2.3**. (Consistency)

- Assertions $a_{i_{1}}\in C_{j_{1}}$ and $a_{i_{2}}\in C_{j_{2}}$ are *consistent* if (1) $a_{i_{1}}\neq a_{i_{2}}$ or (2) $C_{j_{1}}\cap C_{j_{2}}\neq ⊥$
- Assertions $a_{i_{1}}\in C_{j_{1}}$ and $a_{i_{2}}\in C_{j_{2}}$ are *inconsistent* if $a_{i_{1}}=a_{i_{2}}$ and $C_{j_{1}}\cap C_{j_{2}}=⊥$
- A set of assertions $A=\{a_{i_{1}}\in C_{j_{1}},a_{i_{2}}\in C_{j_{2}},...,a_{i_{n}}\in C_{j_{n}}\}$ is *consistent* if for all *k*, *l* ∈ {1, 2, …, *n*}, $a_{i_{k}}\in C_{j_{k}}$ and $a_{i_{l}}\in C_{j_{l}}$ are consistent. Individually consistent sets *A*_1_ and *A*_2_ are consistent with each other if *A*_1_ ∪ *A*_2_ is consistent.

### 3.3 Reasoning

Terminological axioms are expressed as predicated statements, can be used to form new assertional axioms. These statements describe relationships between classes, so once we know that an individual is a member of a class, we can infer its relationship to other classes based on the ontology’s terminological axioms. The new assertional axioms can then be used in new arguments, revealing more axioms. Long chains of reasoning can form in this way. These arguments hinge on the rule of universal instantiation, which states that if something is true in general for all individuals in a class, it is true for each specific individual in that class. For our purposes we express the rule of universal instantiation as: if *C* ⊆ *D* and *a* ∈ *C*, infer *a* ∈ *D*. If *C* ∩ *D* = ⊥ and *a* ∈ *C*, infer *a* ∉ *D*.

### 3.4 Bayesian knowledge bases

Bayesian Knowledge Bases [9, 10] are a generalization of Bayesian Networks. As opposed to BNs, BKBs specify dependence at the instantiation level instead of the random variable level. BKBs allow for cycles between variables, and do not require the complete probability distribution to be specified. BKBs model probabilistic knowledge in an intuitive “if-then” rule structure which quantifies dependencies between states of random variables. Reasoning with BKBs is performed as belief updating, belief revision, or partial relief revision. Belief updating computes the posterior probability of a target variable state, belief revision computes the posterior probabilities of domain instantiations, and partial belief revision computes the posterior probabilities of sets of target variable states. BKBs excel at modeling causal and correlative information because they provide backtraceable explanations of simulation outcomes [35]. They see use on problems such as war gaming [36], predicting outcomes of strategic actions [37], insider threat detection [38], and Bayesian structure learning [39]. Most importantly, unlike BNs, multiple BKB fragments can be combined into a single valid BKB using the BKB fusion algorithm [40]. The idea behind this algorithm is to take the union of all input fragments by incorporating source nodes, which indicate the source and reliability of the fragments. BKB fusion preserves all knowledge and allows for source and contribution analysis to determine the impact of source knowledge on reasoning results.

There are two equivalent formulations of BKB theory. One, presented in Santos et al. (2003) [10], defines a BKB as a set of conditional probability rules (CPRs) and the other, presented in Santos et al. (1999) [9], defines a BKB as a directed graph. In this section, we present a condensed version of the CPR-based formulation. The notation is slightly modified but expresses equivalent concepts.

**Definition 3.4.1**. Let {*A*_1_, …, *A*_*n*_} be a collection of finite discrete random variables (rvs) where *r*(*A*_*i*_) denotes the set of possible values for *A*_*i*_. A *conditional probability rule* is a statement of the form

$$\begin{array}{c} P(A_{i_{n}}=a_{i_{n}}|A_{i_{1}}=a_{i_{1}}\wedge A_{i_{2}}=a_{i_{2}}\wedge ...\wedge A_{i_{n-1}}=a_{i_{n-1}})=p \end{array}$$

for some positive integer *n* where $a_{i_{j}}\in r\left(A_{i_{j}}\right)$ such that *i*_*j*_ ≠ *i*_*k*_ for all *j* ≠ *k* and *p* ∈ [0, 1] is a weight.

A CPR *R*’s antecedent, denoted *ant*(*R*), is the conjunction of rv assignments to the right of the vertical bar. *R*’s consequent, denoted *con*(*R*), is the rv assignment to the left of the vertical bar. *R* states that given the antecedent, the consequent is true with probability *p*. Each rv assignment in the antecedent is called an immediate ancestor of the consequent, and the consequent is called an immediate descendant of the rv assignments in the antecedent. Note that an empty antecedent reflects a prior probability.

**Definition 3.4.2**. Given two CPRs:

$$R_{1}:P\left(A_{i_{n}}=a_{i_{n}}|A_{i_{1}}=a_{i_{1}}\wedge A_{i_{2}}=a_{i_{2}}\wedge ...\wedge A_{i_{n-1}}=a_{i_{n-1}}\right)=p_{1}$$

$$R_{2}:P\left(A_{j_{m}}=a_{j_{m}}^{\prime }|A_{j_{1}}=a_{j_{1}}^{\prime }\wedge A_{j_{2}}=a_{j_{2}}^{\prime }\wedge ...\wedge A_{j_{m-1}}=a_{j_{m-1}}^{\prime }\right)=p_{2}$$

we say that *R*_1_ and *R*_2_ are *mutually exclusive* if there exists some 1 ≤ *k* < *n* and 1 ≤ *l* < *m* such that *i*_*k*_ = *j*_*l*_ and $a_{i_{k}}\neq a_{j_{l}}^{\prime }$. Otherwise, we say they are *compatible*.

Intuitively, the antecedents of mutually exclusive CPRs cannot be simultaneously satisfiable because they are conditioned on different values of the same rv(s).

**Definition 3.4.3**. *R*_1_ and *R*_2_ are *consequent bound* of (1) for all *k* < *n* and *l* < *m*, $a_{i_{k}}=a_{j_{l}}^{\prime }$ whenever *i*_*k*_ = *j*_*l*_ and (2) *i*_*n*_ = *j*_*m*_ but $a_{i_{n}}\neq a_{j_{m}}^{\prime }$

Intuitively, consequent bound CPRs only conflict in their consequent. Their antecedents are compatible, but their consequents assign different values to the same rv. We use mutual exclusivity and consequent boundedness to define a BKB below:

**Definition 3.4.4**. A Bayesian Knowledge Base *B* is a finite set of CPRs such that.

- for any distinct *R*_1_ and *R*_2_ in *B*, either (1) *R*_1_ is mutually exclusive with *R*_2_ or (2) *con*(*R*_1_) ≠ *con*(*R*_2_); and
- for any subset *S* of mutually consequent bound CPRs of *B*, ∑_*R*∈*S*_ *P*(*R*) ≤ 1

The following definitions establish the concept of inferences, which are the basis of a BKB’s expression of probability distributions.

**Definition 3.4.5**. For a CPR

$$\begin{array}{c} R:P(A_{i_{n}}=a_{i_{n}}|A_{i_{1}}=a_{i_{1}}\wedge A_{i_{2}}=a_{i_{2}}\wedge ...\wedge A_{i_{n-1}}=a_{i_{n-1}})=p \end{array}$$

A subset *S* of BKB *B* is said to be a *deductive set* if for each *R* ∈ *S* the following two conditions hold:

- For each *k* = 1, …, *n*−1 there exists a CPR *R*_*k*_ ∈ *S* such that $con\left(R_{k}\right)=\left\{A_{i_{k}}=a_{i_{k}}\right\}$
- There does not exist some *R*′ ∈ *S* where *R*′ ≠ *R* and *con*(*R*′) = *con*(*R*).

The first condition establishes that each rv in *R*’s antecedent must be supported by the consequents of other CPRs. The second condition requires that each rv assignment be supported by a unique set of ancestors.

**Definition 3.4.6**. A deductive set *I* is said to be an *inference* over *B* if *I* consists of mutually compatible CPRs and no rv assignment is an ancestor of itself in *I*. The set of rv assignments induced by *I* is denoted *V*(*I*). The probability of *I* is defined as *P*(*I*) = ∏_*R*∈*I*_ *P*(*R*)

**Definition 3.4.7**. Two inferences are *compatible* if all their CPRs are mutually compatible.

The following theorems establish that inferences can define a partial joint probability distribution. Proofs can be found in [10]

**Theorem 3.4.1**. For each set of rv assignments *V*, there exists at most one inference *I* over *B* such that *V* = *V*(*I*).

**Theorem 3.4.2**. For any set of mutually incompatible inferences *Y* in *B*, ∑_*I*∈*Y*_ *P*(*I*) ≤ 1.

**Theorem 3.4.3**. Let *I*_0_ be some inference. For any set of mutually incompatible inferences *Y*(*I*_0_) such that for all *I* ∈ *Y*(*I*_0_), *I*_0_ ⊆ *I*, ∑_*I*∈*Y*(*I*_0_)_ *P*(*I*) ≤ *P*(*I*_0_)

**Fig 1:**
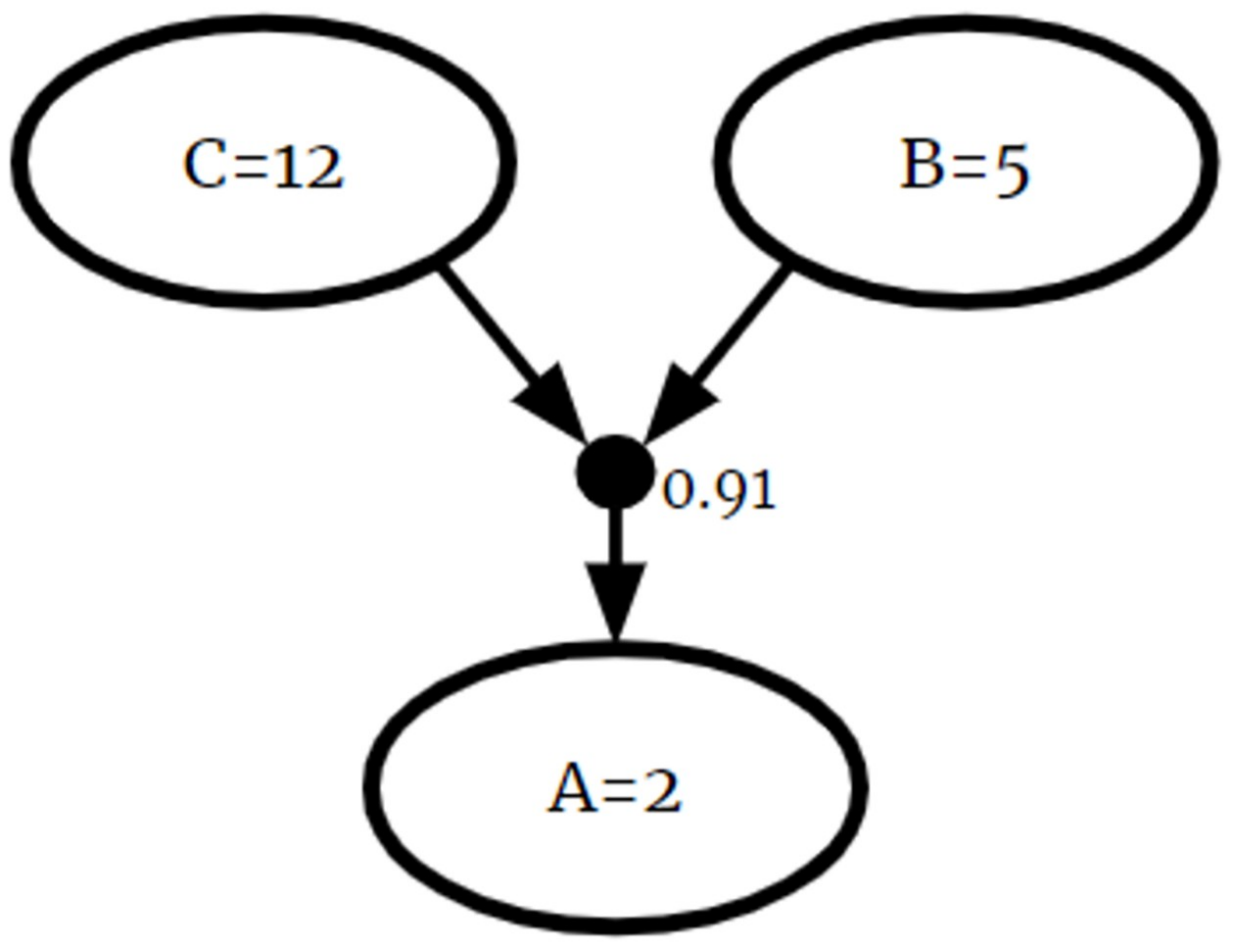
Example CPR. A graphical representation of a CPR.

We used the conditional probability rule formulation of BKBs throughout this paper. However, the directed graph model allows for intuitive visual representations of BKBs. These graphs are comprised of two types of node: instantiation nodes, I-nodes, and support nodes, S-nodes. I-nodes represent random variable instantiations and S-nodes represent the conditional dependencies between them. A weighting function assigns a probability to the CPR represented by each S-node. For example, a graphical representation of the CPR:

$$\begin{array}{c} P(A=2|B=5\wedge C=12)=0.91 \end{array}$$

is shown in Fig 1. In this example, the black node is an S-node and white nodes I-nodes.

**Fig 2:**
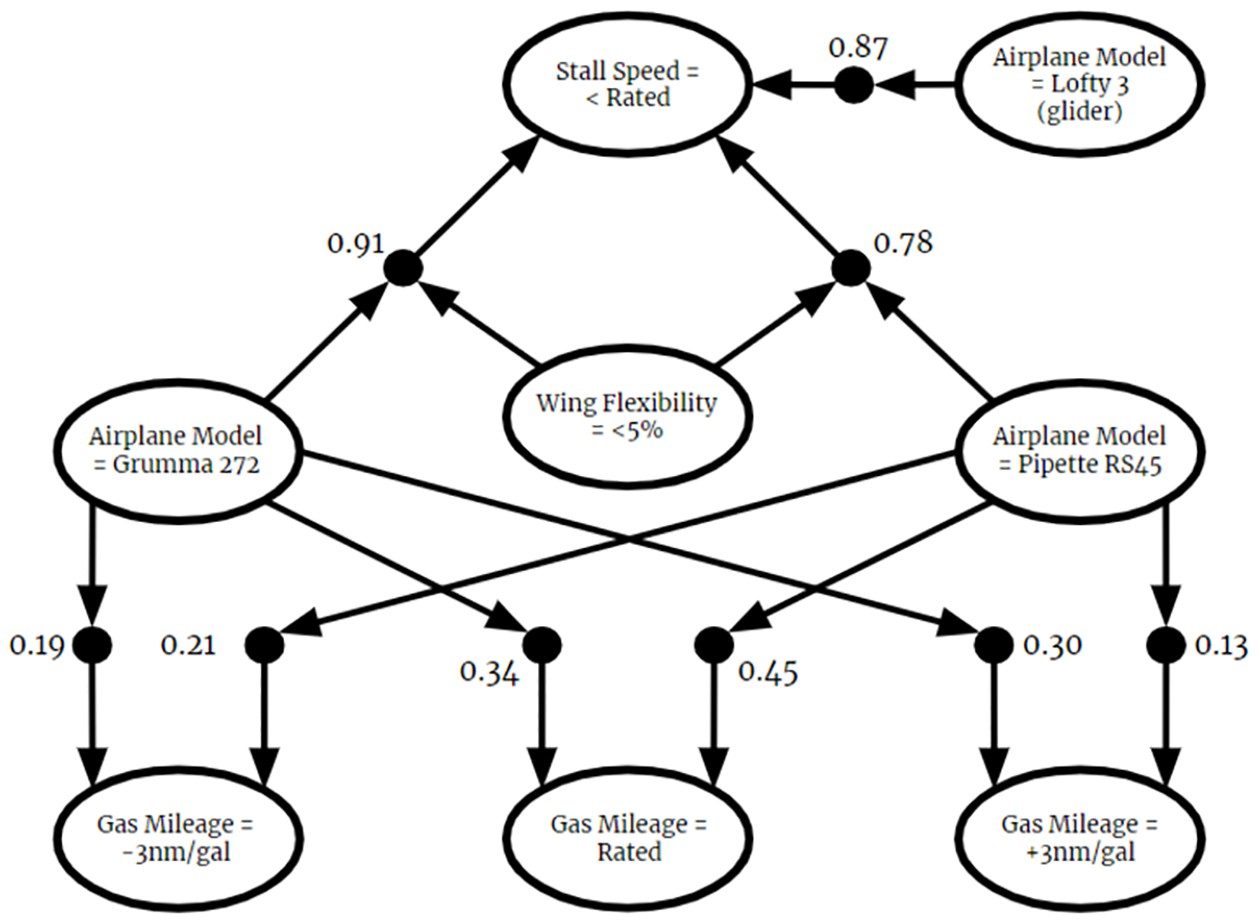
Example BKB. A graphical representation of a BKB.

Many CPRs are combined to form a larger BKB. An example BKB is shown in Fig 2.

### 3.5 Bayesian knowledge fusion

One might want to combine the knowledge represented in BKBs from two or more distinct sources. A BKB fusion algorithm [40] is used to do so. We will summarize BKB fusion in the remainder of this subsection. Consider the two knowledge fragments:

$$F_{1}=\{P(B=b)=0.2,P(A=a|B=b)=0.8\}$$

$$F_{2}=\{P(C=c)=0.4,P(A=a|C=c)=0.35\}$$

These fragments could be naively combined by taking the union of *F*_1_ and *F*_2_ to form *F*_3_:

$$F_{3}=\{P(B=b)=0.2,P(C=c)=0.4,P(A=a|B=b)=0.8,P(A=a|C=c)=0.35\}$$

**Fig 3:**
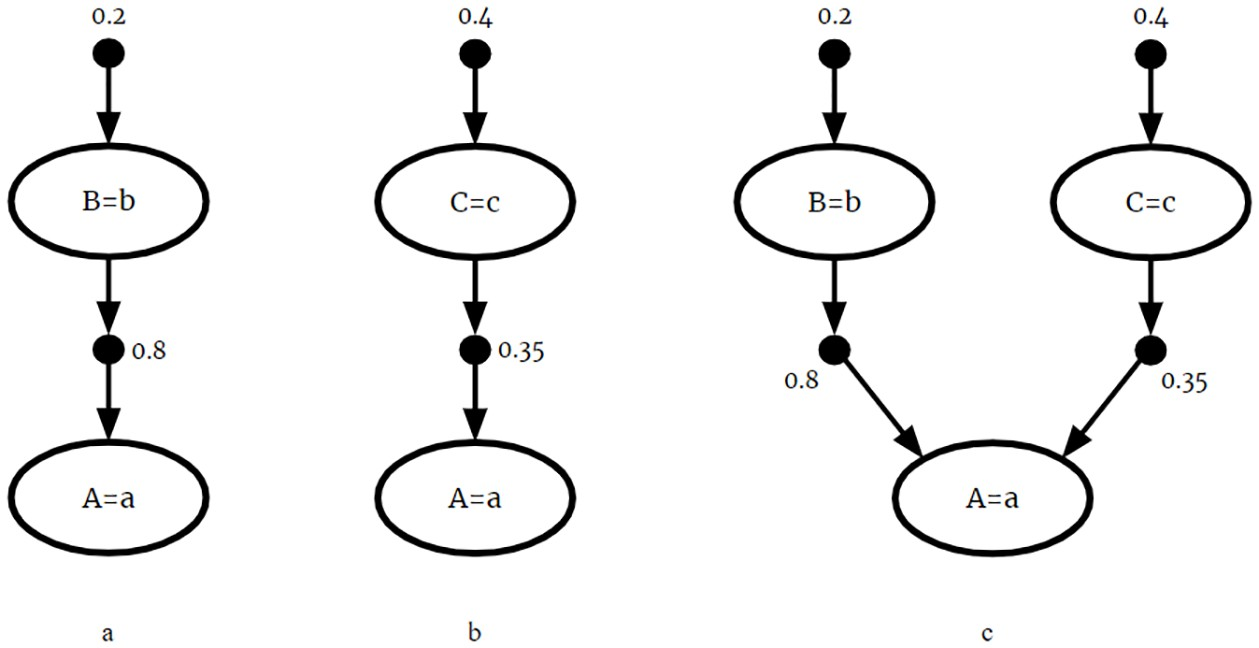
Naive fusion. The naive fusion of (a) *F*_1_ and (b) *F*_2_. Since it is possible *B* = *b* and *C* = *c* simultaneously, (c) naive fusion violates mutual exclusivity.

The CPRs *P*(*A* = *a*|*B* = *b*) and *P*(*A* = *a*|*C* = *c*) have equal consequents, but their antecedents are not mutually exclusive. So this union would violate the mutual exclusivity requirement of BKBs, and the result *F*_3_ is not a valid BKB. This naïve fusion is displayed graphically in Fig 3.

To address this issue, source information is included in the fused BKB as additional CPRs. This source information represents the reliability of each source BKB. The source reliability is often determined by those building the BKB, although it is possible for source reliability to be updated as new evidence is considered. In this example, we will give *F*_1_ and *F*_2_ equal reliability scores of 0.5 each. The fused *F*_3_ with source information is as follows:

$$\begin{array}{l} F_{3}=\{P(S_{B}=1)=0.5,P(S_{C}=2)=0.5,P(S_{A}=1)=0.5,P(S_{A}=2)\\ =0.5,P(B=b|S_{B}=1)=0.2,P(C=c|S_{C}=2)=0.4,P(A=A|B=b\\ \wedge S_{A}=1)=0.8,P(A=a|C=c\wedge S_{A}=2)=0.35\} \end{array}$$

**Fig 4:**
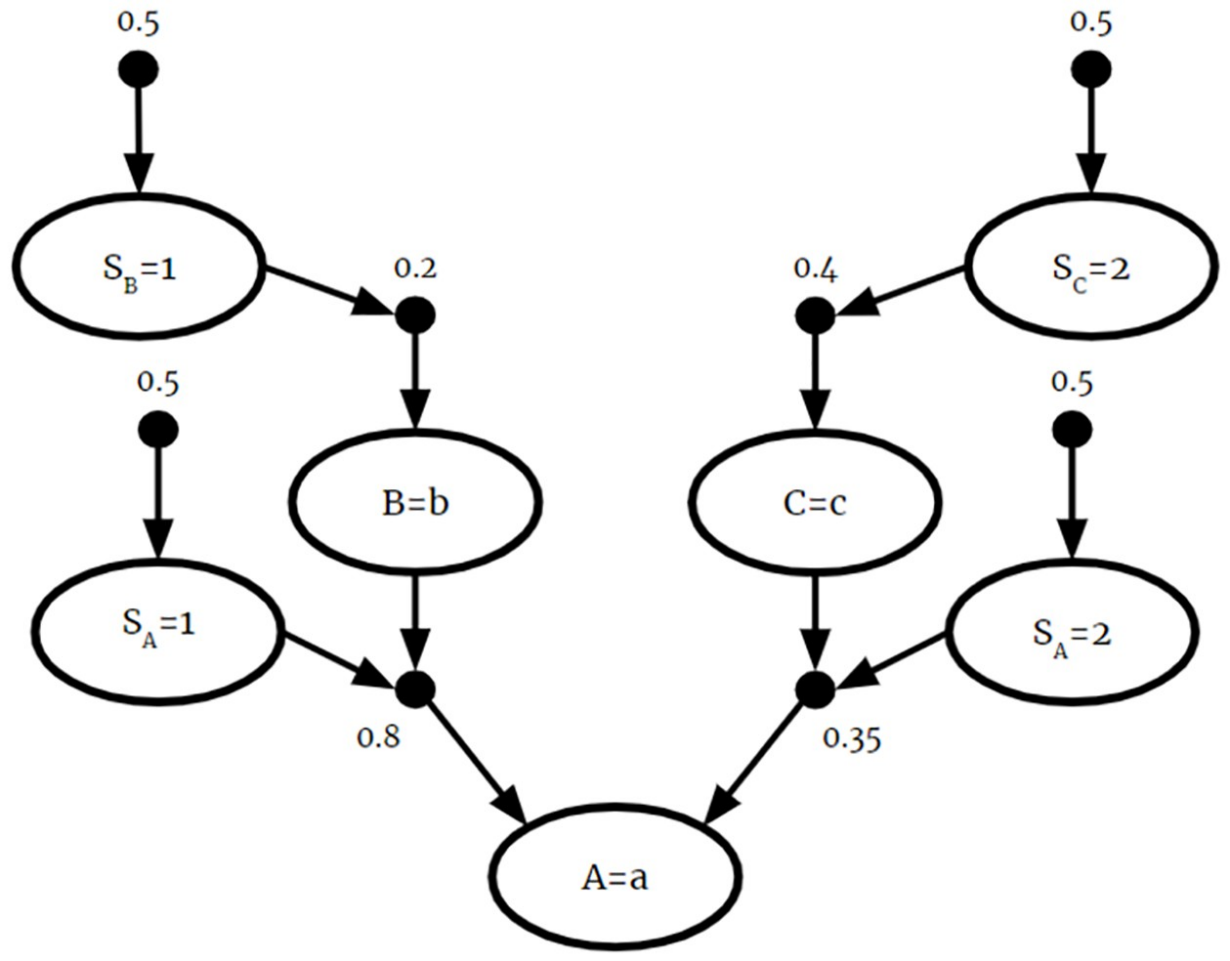
BKB fusion. The fusion of *F*_1_ and *F*_2_. The result is a valid BKB.

By incorporating source information, the fused *F*_3_ is a valid BKB. By including source node *S*_*A*_ in the antecedent of *P*(*A* = *a*|*B* = *b* ∧ *S*_*A*_ = 1) = 0.8 and *P*(*A* = *a*|*C* = *c* ∧ *S*_*A*_ = 2) = 0.35, the two CPRs from different sources are guaranteed to be mutually exclusive. This is graphically represented in Fig 4.

The algorithm to fuse a set of BKB fragments is found in [40]. From [40], we adopt the following theorem:

**Theorem 3.5.1**. The output *K*′ = (*G*′, *w*′) of Bayesian Knowledge Fusion is a valid BKB

**Fig 5:**
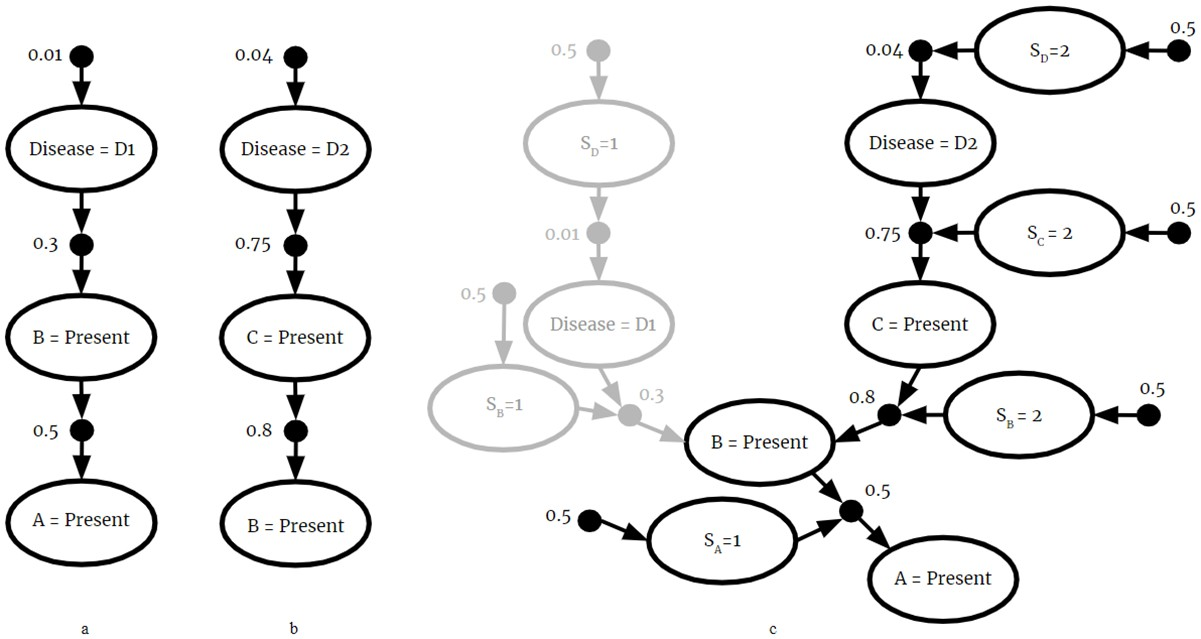
New inference. The fusion of two BKB fragments (a) and (b). The most probable inference in (c) the fused BKB is not present in either (a) or (b).

Perhaps the most useful feature of the fusion algorithm is its ability to discover new inferences which are present in the fused BKB, but not in the input BKBs. Consider the example in Fig 5. We have rvs for symptoms *A*, *B*, and *C* that can either be present in a patient or not. We have another rv representing the disease a patient might have. Assume a patient has symptom *A*. From the given fragments we can only conclude that the patient had disease *d*_1_. Note that we cannot conclude that the patient has disease *d*_2_ because it is not included in fragment 1. Fragment 2 does include *d*_2_ but does not include symptom *A*. However, when the fragments are fused, we find that disease *d*_2_ is most probable. In such ways, fusion can facilitate the discovery of new insights previously unknown to its sources.

## 4 Bayesian knowledge-driven ontologies: Principles and structure

An instantiation of a domain is an assignment of each known individual to known classes. An individual may be assigned to one or more than one class, and a class may be assigned any number of individuals. A BKO models a probability distribution over all of a domain’s possible instantiations and uses if-then rules to restrict and reason about that distribution’s probability mass function (pmf). This gives a user a formal way to reason in detail about relative likelihoods of the domain’s possible states. BKO theory supports incompleteness, so it does not require a complete definition of the pmf. Therefore, a valid BKO may be compatible with more than one pmf. This allows the user to draw valid conclusions from knowledge that would be insufficient for other probabilistic reasoning methods. Furthermore, thanks to its grounding in BKB theory, reasoning can be performed whether the BKO is consistent or not. Checking for probabilistic consistency has historically been a challenge among uncertain semantic network formalisms, but is not a requirement for BKOs.

To formulate this theory, we first define the nature of this probability distribution in terms of its sample space and random variables. We then define the means of expressing knowledge in BKO theory, which is done by declaring probabilistic if-then relationships between variables. Finally, we define the structure of a BKO as a knowledge base, and its mapping to its close cousin the BKB, leading into Section 5 on the reasoning that can be performed with a BKO.

### 4.1 Model of a domain

Recall our first point from the introduction: uncertainty is the presence of multiple possible states of the world, such that we have insufficient knowledge to determine which state is true but can still define a probability distribution over its possible states. This is commonly referred to as “distribution semantics”. The following definitions describe our implementation of distribution semantics for BKO theory.

**Definition 4.1.1**. For a domain *Q*, a finite set of individuals *I*, and a finite set of classes *C*, a lexicon *L*(*Q*) = *I* × *C*.

**Notation**. Use the notation *I*(*Q*) and *C*(*Q*) as functions to access *I* and *C* independently.

**Definition 4.1.2**. A set of assertions $A=\{a_{i_{1}}\in C_{j_{1}},a_{i_{2}}\in C_{j_{2}},...,a_{i_{n}}\in C_{j_{n}}\}$ with *L*(*A*) = *L*(*Q*) is an *instantiation* of *Q* only if:

- *A* is consistent
- A contains no terminological knowledge
- For every *a*_*l*_ ∈ *I* and *C*_*k*_ ∈ *C*, either *a*_*l*_ ∈ *C*_*k*_ or *a*_*l*_ ∈ ¬*C*_*k*_

**Notation**. For a domain *Q* comprised of individuals {*a*_1_, *a*_2_, … *a*_*m*_} and classes {*C*_1_, *C*_2_, … *C*_*n*_} denote Ω(*Q*) as the set of all possible instantiations of *Q*, where

$$\begin{array}{c} \Omega \left(Q\right)=\prod_{i=1}^{m}\prod_{j=1}^{n}\left\{a_{i}\in C_{j},a_{i}\in \neg C_{j}\right\} \end{array}$$

Note that in practice, one will never generate a full instantiation of a domain, but it is a fundamental concept of the theory.

**Definition 4.1.3**. Let *f* : Ω(*Q*) → [0, 1] be a probability distribution for domain *Q*. This is known as the domain’s *state distribution*.

### 4.2 Asserting knowledge

In BKO theory, knowledge is asserted by declaring if-then conditional probability rules between variables. There are two types of rules used, probabilistic assertional axioms and probabilistic terminological axioms. Probabilistic assertional axioms are propositional, they characterize a single individual’s conditional probability of membership in a class. Probabilistic terminological axioms are predicated, or first-order. They implicitly define conditional probabilities of class membership for unspecified individuals. In Section 5 we define how these implicit probabilities can used to create probabilistic assertional axioms.

**Definition 4.2.1**. A set of classes {*C*_1_, …, *C*_*n*_} is said to *partition* a class *D* if $\cup_{i=1}^{n}C_{i}=D$ and for any *C*_*i*_, *C*_*j*_ ∈ {*C*_1_, …, *C*_*n*_}, *C*_*i*_ ∩ *C*_*j*_ = ⊥.

**Proposition 4.2.1**. Let *C* = {*C*_1_, …, *C*_*n*_} be a set of classes that partition *D* and *a* be an individual. Then there exists a random variable *V* such that *r*(*V*) = {*a* ∈ *C*_1_, …, *a* ∈ *C*_*n*_}.

Proposition 4.2.1 is crucial for the remaining sections. Later we discuss how to instantiate terminological knowledge. The insight that a random variable is induced for an individual that is a member of a set of disjoint classes allows us to do so.

**Definition 4.2.2**. A *probabilistic assertional axiom* (PAA) is a conditional probability rule of the form:

$$\begin{array}{c} R:P(V_{i_{n}}=\left\{a_{i_{n}}\in C_{j_{n}}\right\}|V_{i_{1}}=\left\{a_{i_{1}}\in C_{j_{1}}\right\}\wedge ...\wedge V_{i_{n-1}}=\left\{a_{i_{n-1}}\in C_{j_{n-1}}\right\})=p \end{array}$$

Such that $\left\{a_{i_{k}}\in C_{j_{k}}\right\}\in r\left(V_{i_{k}}\right)$, $\left\{a_{i_{1}}\in C_{j_{1}},...,a_{i_{n}}\in C_{j_{n}}\right\}$ is consistent and there exists exactly one individual $a_{i_{k}}$ in any assertion $R\in r(V_{i_{k}})$.

A PAA R’s antecedent, denoted ant(R), is the conjunction of random variables to the right of the vertical bar. R’s consequent, denoted con(R), is the random variable assignment to the left of the vertical bar. In this case $ant\left(R\right)=V_{i_{1}}=\left\{a_{i_{1}}\in C_{j_{1}}\right\}\wedge ...\wedge V_{i_{n-1}}=\left\{a_{i_{n-1}}\in C_{j_{n-1}}\right\}$ and $con\left(R\right)=V_{i_{n}}=\left\{a_{i_{n}}\in C_{j_{n}}\right\}$. The notation used to represent PAAs, R, was chosen to reflect that PAAs are CPRs, which are denoted *R* in BKBs.

The other rule used in BKO theory is the probabilistic terminological axiom. However, its definition relies on variable individuals and variable concept constructors, so we must define those first.

**Definition 4.2.3**. A *variable individual* is a variable $\hat{x}$ which represents an unspecified *a* ∈ *I*(*Q*) We will use the term *specific individual* to distinguish a normal individual from a variable individual.

**Definition 4.2.4**. A *variable concept* is a concept $\hat{C}$ whose members include one or more variable individuals. We will use the term *specific concept* to distinguish a concept from a variable concept.

**Definition 4.2.5**. Let $\hat{X}=\left\{\hat{x_{1}},\hat{x_{2}},...,\hat{x_{n}}\right\}$ be a set of variable individuals and *L*(*Q*) be a lexicon describing domain *Q*. A *variable concept constructor* is a function $f(L\left(Q\right),\hat{X})$, the output of which is a variable concept.

**Definition 4.2.6**. A *variable assertion* is an assertion of the form $\hat{y}\in \hat{C}$ where $\hat{y}$ is a variable individual and $\hat{C}$ is either variable concept or specific concept.

**Notation**. Letters with a hat (^^^) represent variable individuals or concepts, while letters without a hat (^^^) represent specific individuals, classes, roles, etc.

For example, the variable concept $\hat{C}=R_{1}\left(\hat{y},\hat{x}\right)\cap R_{2}\left(\hat{y},\hat{x}\right)$ represents some variable individual $\hat{y}$ being related to some yet-unspecified individual $\hat{x}$ by both properties *R*_1_ and *R*_2_. Note that variable concepts are permitted to contain some specific individuals too. $\hat{C}=R_{1}\left(\hat{y},\hat{x}\right)\cap R_{2}\left(\hat{y},\hat{x}\right)\cap R_{3}\left(\hat{y},b\right)$ represents being related to yet-unspecified individual $\hat{x}$ by *R*_1_ and *R*_2_, and to specific individual *b* by *R*_3_.

**Definition 4.2.7**. For a set of variable individuals $\left\{{\hat{x}}_{1},...,{\hat{x}}_{n}\right\}$ and a set of variable concepts $\left\{{\hat{C}}_{1},...,{\hat{C}}_{n}\right\}$ a *probabilistic terminological axiom* (PTA) is a statement of the form

$$\begin{array}{c} T:P({\hat{x}}_{i_{n}}\in {\hat{C}}_{j_{n}}|{\hat{x}}_{i_{1}}\in {\hat{C}}_{j_{1}}\wedge ...\wedge {\hat{x}}_{i_{n-1}}\in {\hat{C}}_{j_{n-1}})=p \end{array}$$

such that for some *k* ≠ *n*, either (1) *i*_*k*_ = *i*_*n*_, (2) ${\hat{C}}_{j_{k}}$ contains ${\hat{x}}_{i_{n}}$ in its formula, or both.

As with PAAs, a PTA’s antecedent and consequent are the terms to the right and left of the vertical bar. Note that not all members of a PTA’s antecedent must be variable assertions. There must be at least one due to the requirement that the individual in its consequent must be defined in the antecedent.

PTAs are a first-order generalization of the strictly propositional PAA. They facilitate forming complex universal quantification statements, which lets BKO theory express advanced DL notions like property attributes. In fact, BKO theory can be used to express complex custom property attributes not available in DL. A more intuitive explanation is best communicated through some examples. Start with the simplest form of a PTA:

$$\begin{array}{c} T:P(\hat{x}\in C_{2}|\hat{x}\in C_{1})=p \end{array}$$

This expresses that any member of *C*_1_ has a probability *p* of also being a member of *C*_2_. PTAs are also a mechanism for expressing complex probabilistic rules extending some of the features of more advanced forms of DL. In the following example, let *R* be a specific relational property.

$$\begin{array}{c} T:P(\hat{x}\in R\left(\hat{x},\hat{y}\right)|\hat{y}\in R\left(\hat{y},\hat{x}\right))=p \end{array}$$

This PTA can be read as “The probability that any *x* is related to any *y* by *R* given any *y* is related to any *x* by *R*”. Should *p* = 1, *T*_1_ would declare *R* to be a symmetric property. Similarly, the PTA

$$\begin{array}{c} T:P(\hat{x}\in R\left(\hat{x},\hat{z}\right)|\hat{x}\in R\left(\hat{x},\hat{y}\right)\wedge R\left(\hat{y},\hat{z}\right))=p \end{array}$$

would declare *T* to be a transitive property, should *p* = 1. Note that *p* does not necessarily need to be equal to one. Consider the following PTA:

$$\begin{array}{c} T:P(\hat{x}\in R\left(\hat{x},\hat{x}\right)|\hat{x}\in C)=p \end{array}$$

With *p* = 1, *T* becomes a reflexive property. But if we set *p* = 0.7, *T* states that for any individual $\hat{x}$ in class *C*, there is a 0.7 chance that it is related to itself by property *R*. It should be apparent that we can go beyond the offerings of DL to create much more sophisticated terminological expressions.

A PTA must eventually be instantiated, a process that assigns each one of a PTA’s variable individuals to a specific individual, resulting in a PAA.

**Definition 4.2.8**. Let *X* = {*x*_1_, *x*_2_, …, *x*_*w*_} be a set of specific individuals and $\hat{X}=\left\{{\hat{x}}_{1},{\hat{x}}_{2},...,{\hat{x}}_{w}\right\}$ be a set of variable individuals whose range is *X*. An *instantiation function* $g:\hat{X}\to X$ is a one-to-one mapping of each variable individual to a specific individual.

Note that the instantiation function defined here is the probabilistic counterpart of the interpretation function in classic DL.

**Notation**. For some expression *E* and an instantiation function *g*, *E* instantiated by *g* may be written as either *g*(*E*) or *E*|_*g*_. So the concept constructor $\hat{C}=f\left(L\left(Q\right),\left\{{\hat{x}}_{1},...,{\hat{x}}_{w}\right\}\right)$ evaluated by *g* could be written $\hat{C}{{|}_{g}=f\left(L\left(Q\right),\left\{{\hat{x}}_{1},...,{\hat{x}}_{w}\right\}\right)|}_{g}=g\left(f\left(L\left(Q\right),\left\{{\hat{x}}_{1},...,{\hat{x}}_{w}\right\}\right)\right)$

**Proposition 4.2.2**. $\hat{C}{|}_{g}=g\left(f\left(L\left(Q\right),\left\{{\hat{x}}_{1},...,{\hat{x}}_{w}\right\}\right)\right)=f\left(L\left(Q\right),\left\{g\left({\hat{x}}_{1}\right),...,g\left({\hat{x}}_{w}\right)\right\}\right))$

**Definition 4.2.9**. The *instantiation* of a PTA

$$\begin{array}{c} T:P({\hat{x}}_{i_{n}}\in {\hat{C}}_{j_{n}}|{\hat{x}}_{i_{1}}\in {\hat{C}}_{j_{1}}\wedge ...\wedge {\hat{x}}_{i_{n-1}}\in {\hat{C}}_{j_{n-1}})=p \end{array}$$

by instantiation function $g\left(\left\{{\hat{x}}_{1},...,{\hat{x}}_{n}\right\}\right)=\left\{a_{1},...,a_{n}\right\}$ is tha PAA

$$\begin{array}{c} {T|}_{g}:P\left(V_{i_{n}}=\left\{a_{i_{n}}\in C_{j_{n}}\right\}|V_{i_{1}}=\left\{a_{i_{1}}\in C_{j_{1}}\right\}\wedge ...\wedge V_{i_{n-1}}=\left\{a_{i_{n-1}}\in C_{j_{n-1}}\right\}\right)=p \end{array}$$

*T*|_*g*_ may be read “*T* evaluated by *g*.” For a simple PAA like $T:P(\hat{x}\in C_{2}|\hat{x}\in C_{1})=p$ and instantiation function $g(\hat{x})=a$, *T*|_*g*_ can be read “*T* evaluated with $\hat{x}$ equal to *a*”. Note that the probability value assigned to the instantiated PTA is the same as it was before being instantiated. This is what is meant when we say that PTAs describe a pmf. Unlike PAAs, PTAs on their own are not conditional probability rules. PTAs themselves do not have an effect on the pmf but any PAA that is an instantiation of them does.

PTAs and PAAs are flexible enough to represent classical axioms. For example a classical assertional axiom *Z* is equivalent to the unconditional PAA *P*(*Z*) = 1. A subsumption axiom *C* ⊆ *D* is equivalent to the PTA $P(\hat{x}\in D|\hat{x}\in C)=1$, and a disjointness axiom *C*∩*D* = ⊥ is equivalent the PTAs $P(\hat{x}\in D|\hat{x}\in C)=0$ and $P(\hat{x}\in C|\hat{x}\in D)=0$.

### 4.3 Logical and probabilistic consistency

We will now develop the constraints necessary to guarantee that a BKO induces a valid probability mass function. These definitions will parallel those of BKB theory. First we define mutual exclusivity and consequent boundedness in PAAs and PTAs. These definitions will be analogous to their respective concepts from BKB theory, Definitions 3.4.2 and 3.4.3. Let

$$R_{1}:P\left(V_{i_{n}}=\left\{a_{i_{n}}\in C_{j_{n}}\right\}|V_{i_{1}}=\left\{a_{i_{1}}\in C_{j_{1}}\right\}\wedge ...\wedge V_{i_{n-1}}=\left\{a_{i_{n-1}}\in C_{j_{n-1}}\right\}\right)$$

$$R_{2}:P\left(V_{k_{m}}=\left\{a_{k_{m}}^{\prime }\in C_{l_{m}}^{\prime }\right\}|V_{k_{1}}=\left\{a_{k_{1}}^{\prime }\in C_{l_{1}}^{\prime }\right\}\wedge ...\wedge V_{k_{m-1}}=\left\{a_{k_{m-1}}^{\prime }\in C_{l_{m-1}}^{\prime }\right\}\right)$$

be two PAAs.

**Definition 4.3.1**. Let *V*_1_ and *V*_2_ be random variables whose sample space is a set of assertions. The instantiations *V*_1_ = {*a*_1_ ∈ *C*_1_} and *V*_2_ = {*a*_2_ ∈ *C*_2_} are *consistent* if {*a*_1_ ∈ *C*_1_} and {*a*_2_ ∈ *C*_2_} are consistent. Otherwise, they are *inconsistent*. Sets of instantiated rvs are consistent if all their members are consistent.

**Definition 4.3.2**. Let $V_{1}=\left\{V_{1_{1}},...,V_{1_{n}}\right\}$ and $V_{2}=\left\{V_{2_{1}},...,V_{2_{m}}\right\}$ be sets of random variables whose sample space is a set of assertions. *V*_1_ and *V*_2_ are *consistent* if for all $V_{1_{i}}\in V_{1}$ and $V_{2_{j}}\in V_{2}$, $V_{1_{i}}$ and $V_{2_{j}}$ are consistent.

We had already defined what it means for assertions and sets of assertions to be consistent. Since PAAs are CPRs and not sets of assertions, This definition is necessary before we can define mutual exclusivity and consequent boundedness for PTAs and PAAs.

**Definition 4.3.3**. The *disaggregation* of a conjunction of assertions *A*_1_ ∧ *A*_2_ ∧ … ∧ *A*_*n*_ is a set of the individual assertions of the conjunction, {*A*_1_, *A*_2_, …*A*_*n*_}, denoted *disag*(*A*_1_ ∧ *A*_2_ ∧ … ∧ *A*_*n*_) = {*A*_1_, *A*_2_, …*A*_*n*_}.

**Definition 4.3.4**. (Mutually Exclusive)

- PAAs $R_{1}$ and $R_{2}$ are *mutually exclusive* if $disag(ant\left(R_{1}\right))$ is inconsistent with $disag(ant\left(R_{2}\right))$.
- PTAs *T*_1_ and *T*_2_ are *mutually exclusive* if $T_{1}{|}_{g_{1}}$ and $T_{2}{|}_{g_{2}}$ are mutually exclusive for any instantiation functions *g*_1_ and *g*_2_. Recall that the instantiation of a PTA is a PAA.
- A PAA R and PTA *T* are *mutually exclusive* if there exists some instantiation function *g* such that R and *T*|_*g*_ are mutually exclusive.

**Definition 4.3.5**. (Consequent Bound)

- PAAs $R_{1}$ and $R_{2}$ are *consequent bound* if $disag(ant\left(R_{1}\right))$ is consistent with $disag(ant\left(R_{2}\right))$ but $con(R_{1})$ and $con(R_{2})$ are inconsistent.
- PTAs *T*_1_ and *T*_2_ are *consequent bound* if $T_{1}{|}_{g_{1}}$ and $T_{2}{|}_{g_{2}}$ are consequent bound for any instantiation functions *g*_1_, *g*_2_.
- A PAA R and PTA *T* are *consequent bound* if there exists some instantiation function *g* such that R and *T*|_*g*_ are consequent bound.

**Notation**. The negation of an assertion *a* ∈ *C* is the assertion *a* ∈ ¬*C*

**Definition 4.3.6**. A *Bayesian Knoweldge-driven Ontology*, *B*, is a finite set of PAAs and PTAs such that:

- For any distinct PAAs $R_{1},R_{2}\in B$, either (1) $R_{1}$ and $R_{2}$ are mutually exclusive or (2) $con(R_{1})$ is consistent with the negation of $con(R_{2})$ and $con(R_{2})$ is consistent with the negation of $con(R_{1})$.
- For any distinct PTAs *T*_1_, *T*_2_ ∈ *B* and instantiation functions *g*_1_ and *g*_2_, either (1) $T_{1}{|}_{g_{1}}$ and $T_{2}{|}_{g_{2}}$ are mutually exclusive or (2) $con(T_{1}{|}_{g_{1}})$ is consistent with the negation of $con(T_{2}{|}_{g_{2}})$ and $con(T_{2}{|}_{g_{2}})$ is consistent with the negation of $con(T_{1}{|}_{g_{1}})$.
- For any PAA R and PTA *T* in *B* such that con(R)=a∈C and $con\left(T\right)=\hat{x}\in \hat{D}$, and instantiation function *g*, either (1) R and *T*|_*g*_ are mutually exclusive, (2) con(R) is consistent with the negation of *con*(*T*|_*g*_) and *con*(*T*|_*g*_) is consistent with the negation of con(R), or (3) $T{|}_{g}=R$
- For any subset *S* ⊆ *B* where the PAAs R⊆S and PTAs *T* ⊆ *S* are mutually consequent bound, ∑_*Q*∈*S*_ *P*(*Q*) ≤ 1

**Proposition 4.3.1**. Any subset of a BKO is also a BKO

Definition 4.3.6 has some seemingly odd conditions of a consequent’s consistency with another consequent’s negation. These conditions exist to prevent conflicts between CPRs which are not mutually exclusive but would generate mutex violations in rules mandated by DL. For example, if $con(R_{1})$ said “*a* is in *C*”, but $con(R_{2})$ said “*a* is *D*”, where *D* ⊆ *C*, the laws of any governing DL would require the a PAA $R_{3}$ to be inferred saying “if *a* is in a subset of *C*, then *a* is in *C*”. Without the conditions set in Definition 4.3.6, $R_{3}$ could violate mutex with $R_{1}$. The consequent consistency conditions will catch $R_{1}$ and $R_{2}$ before that inference is computed. Checking whether a set of PAAs and PTAs obeys Definition 4.3.6 requires performing *O*(|*B*|^2^), where |*B*| is the number of PAAs and PTAs in the set.

## 5 BKO reasoning

Recall the purpose of BKO reasoning from the introduction: to determine the posterior probability of some event from the collection of prior and conditional probabilities that constitute our knowledge base. This section defines that process and provides an algorithm outline.

### 5.1 Logical reasoning under uncertainty

Before reasoning, a BKO contains both explicit restrictions on its pmf, in the form of PAAs, and implicit descriptions of its pmf, in the form of the PTAs. The probabilistic rule of universal instantiation is used to convert PTAs to PAAs that restrict the BKO’s pmf.

**Definition 5.1.1**. An assertional axiom *A* is said to be *provable* given a set of assertional and/or terminological axioms *S* iff (1) *A* and *S* are expressible in a governing DL and (2) that governing DL supports a sound algorithm by which *A* given *S* may be proven.

**Definition 5.1.2**. For some provable rule R in the context of some BKO *B*, to *infer* R is to set B←B∪R

**Definition 5.1.3**. For a BKO *B*, a PTA *T* ∈ *B*, and an instantiation function *g*, infer *T*|_*g*_. We call this the *probabilistic rule of universal instantiation*.

**Theorem 5.1.1**. For a BKO *B*, a PTA *T* ∈ *B*, and an instantiation function *g*, *B*∪*T*|_*g*_ is a BKO.

*Proof*. Let *B* be a BKO, *T* ∈ *B* be a PTA, and *g* be an instantiation function. We will show that the finite set of PAAs and PTAs, *B* ∪ *T*|_*g*_ satisfies the four conditions set in the definition of a BKO.

i Since *T* ∈ *B*, condition (iii) holds for *T* and all PAAs $R_{B}\in B$. So, for PAA *T*|_*g*_ and any $R_{B}\in B$ such that ${T|}_{g}\neq R_{B}$, either (1) *T*|_*g*_ and $R_{B}$ are mutually exclusive or (2) *con*(*T*|_*g*_) is consistent the negation of $con(R_{B})$ and $con(R_{B})$ is consistent with the negation of *con*(*T*|_*g*_). Since *B* is a BKO, condition (i) holds for all other PAAs $R_{B_{1}},R_{B_{2}}\in B$. So condition (i) holds for *B*∪*T*|_*g*_.
ii Since *T*|_*g*_ is a PAA, no PTAs were added to *B*. Since *B* is a BKO, all PTAs in *B* satisfy condition (ii).
iii Since *T* ∈ *B*, condition (ii) holds for *T* and all PTAs *T*_*B*_ ∈ *B*. So, for PAA *T*|_*g*_ and any PTA *T*_*B*_ ∈ *B*, either (1) *T*|_*g*_ and $T_{B}{|}_{g_{B}}$ are mutually exclusive or (2) *con*(*T*|_*g*_) is consistent with the negation of $con(T_{B}{|}_{g_{B}})$ and $con(T_{B}{|}_{g_{B}})$ is consistent with the negation of *con*(*T*|_*g*_), or (3) $T_{B}{|}_{g_{B}}=T{|}_{g}$. Since *B* is a BKO, condition (iii) holds for any other PAA $R_{B}$, and PTA *T*_*B*_ in *B*. So then condition (iii) holds for *B* ∪ *T*|_*g*_.
iv Let ⊆ *B* ∪ *T*|_*g*_ be a subset of PAAs and PTAs. **Case 1:** If *T*|_*g*_ ∉ *S* then *S* ⊆ *B*. And since *B* is a BKO, ∑_*Q*∈*S*_ *P*(*Q*) ≤ 1. **Case 2:** If *T*|_*g*_ ∈ *S* then *S* − {*T*|_*g*_} ⊆ *B*, and $\sum_{Q\in S-\{T{|}_{g}\}}$ *P*(*Q*) ≤ 1. But since *T*|_*g*_ is consequent bound with all *Q* ∈ *S* − {*T*|_*g*_}, *T* is also consequent bound with all *Q* ∈ *S* − {*T*|_*g*_}. So there exists a set *S* − {*T*|_*g*_} ∪ *T* ⊆ *B* of mutually consequent bound PAAs and PTAs. and since *B* is a BKO, $\sum_{Q\in S-\{T{|}_{g}\}\cup T}$ *P*(*Q*) ≤ 1. And since PTA *T* and PAA *T*|_*g*_ have the same probability, $\sum_{Q\in S-\{T{|}_{g}\}\cup T}\;P(Q)=\sum_{Q\in S}\;P(Q)\leq 1$

So *B* ∪ *T*|_*g*_ is a finite set of PAAs and PTAs that satisfy the conditions set in Definition 4.3.6. So *B* ∪ *T*|_*g*_ is a BKO.

The goal of a BKO is to express all knowledge as a set of PAAs. One way to guarantee this is by instantiating each PTAs using every possible instantiation function, but this would be computationally impractical. Instead, in advance we identify the combinations of PTAs and instantiation functions that can be used in reasoning.

**Definition 5.1.4**. A PAA R in BKO *B* is *supported* if, for all rv assignments $V_{i}\in ant\left(R\right)$, $V_{i}=con\left(R_{j}\right)$ for some PAA $R_{j}\in B$. Otherwise, R is unsupported. R is *supported by* a set of PAAs {*S*_1_, …, *S*_*n*_} ⊂ *B* if $\left\{con\left(S_{1}\right),...,con\left(S_{n}\right)\right\}=disag\left(ant\left(R\right)\right)$.

**Definition 5.1.5**. A PAA R is said to be *grounded* if (1) ant(R)=∅ or (2) there exists a set of PAA’s *S* = {*S*_1_, *S*_2_, …*S*_*n*_} such that *S* supports R.

Intuitively, grounded PAAs are known pieces of the BKO’s pmf, while ungrounded PAAs are unknown, since they have unknown antecedents. The marginal and posterior probabilities of an ungrounded PAA cannot be computed, so any descendant of that PAA also cannot be computed.

**Proposition 5.1.1**. Let *B* be a BKO and R∈B be an ungrounded PAA. Then (1) any marginal or posterior probabilities computed using the pmf induced by B are identical to those computed using the pmf induced by B-R, and (2) any marginal or posterior probabilities which are incalculable using the pmf induced by *B* are also incalculable using the pmf induced by B-R.

Since ungrounded PAAs do not contribute to a BKO’s pmf, we develop the following notion.

**Definition 5.1.6**. A BKO *B* is *fully-instantiated* when, for any PTA *T* ∈ *B* and instantiation function *g*, either *T*|_*g*_ ∈ *B* or *T*|_*g*_ would not be grounded if added to *B*.

Note that we do not instantiate on infinite numbers of individuals or on unknown individuals. We only work with defined individuals but admit that more are possible per the open-world assumption. A fully instantiated BKO maximizes the number of its supported PAAs. Since the PTAs that could be instantiated to form supported PAAs have been, they are considered redundant in a fully instantiated BKO. However, should new information be added to the BKO, the PTAs would no longer be considered redundant until the BKO was fully instantiated again with the new information.

### 5.2 Mapping a BKO to an equivalent BKB

Recall that PAAs are conditional probability rules, so a set of PAAs constitute a BKB if they satisfy Definition 3.4.4. We will show that a BKO’s A-box is a valid BKB. Furthermore, if a BKO is fully instantiated, no additional information can be inferred from its T-box. Combining these two insights allows us to conclude that a valid BKO can be converted to an equivalent, valid, BKB. We will then be able to use previously developed methods for BKB reasoning. Let

$$R_{1}:P\left(V_{i_{n}}=\left\{a_{i_{n}}\in C_{j_{n}}\right\}|V_{i_{1}}=\left\{a_{i_{1}}\in C_{j_{1}}\right\}\wedge ...\wedge V_{i_{n-1}}=\left\{a_{i_{n-1}}\in C_{j_{n-1}}\right\}\right)=p_{1}$$

$$R_{2}:P\left(V_{k_{m}}=\left\{a_{k_{m}}^{\prime }\in C_{l_{m}}^{\prime }\right\}|V_{k_{1}}=\left\{a_{k_{1}}^{\prime }\in C_{l_{1}}^{\prime }\right\}\wedge ...\wedge V_{k_{m-1}}=\left\{a_{k_{m-1}}^{\prime }\in C_{l_{m-1}}^{\prime }\right\}\right)=p_{2}$$

be two PAAs.

**Lemma 5.2.1**. If $R_{1}$ and $R_{2}$ are mutually exclusive PAAs, then they are mutually exclusive CPRs.

*Proof*. Let $R_{1}$ and $R_{2}$ be two mutually exclusive PAAs. Then $disag(ant\left(R_{1}\right))$ and $disag(ant\left(R_{2}\right))$ are inconsistent, so there exists some *p*, 1 ≤ *p* < *n*, and some *q*, 1 ≤ *q* < *m*, such that *i*_*p*_ = *k*_*q*_ and $a_{i_{p}}=a_{k_{q}}^{\prime }$ but $C_{j_{p}}\cap C_{l_{q}}^{\prime }=⊥$ Then $C_{j_{p}}\neq C_{l_{q}}^{\prime }$, so $V_{h_{p}}=V_{u_{q}}$ but their assignments are not equal. So $R_{1}$ and $R_{2}$ are CPRs that contain the same random variable in their antecedent, but they have different assignments. So $R_{1}$ and $R_{2}$ are mutually exclusive CPRs.

**Lemma 5.2.2**. If $R_{1}$ and $R_{2}$ are consequent-bound PAAs then they are consequent-bound CPRs.

*Proof*. Let $R_{1}$ and $R_{2}$ be two consequent bound PAAs. To show that they are consequent bound CPRs we must show that (1) for all *p* < *n* and all *q* < *m*, $\left\{a_{i_{p}}\in C_{j_{p}}\right\}=\left\{a_{k_{q}}^{\prime }\in C_{l_{q}}^{\prime }\right\}$ whenever *i*_*p*_ = *k*_*q*_, and (2) *i*_*n*_ = *k*_*m*_ but $\left\{a_{i_{n}}\in C_{j_{n}}\right\}\neq \left\{a_{k_{m}}^{\prime }\in C_{l_{m}}^{\prime }\right\}$.

(1) Since $R_{1}$ and $R_{2}$ are consequent bound PAAs, $disag(ant\left(R_{1}\right))$ and $disag(ant\left(R_{2}\right))$ are consistent. So for all *p* < *n* and *q* < *m* whenever *i*_*p*_ = *k*_*q*_, $C_{j_{p}}\cap C_{l_{q}}^{\prime }\neq ⊥$. But since $V_{i_{p}}=V_{k_{q}}$, any classes involved in their assertions are either the equal or disjoint. And since $C_{j_{p}}\cap C_{l_{q}}^{\prime }\neq ⊥$, $C_{j_{p}}=C_{l_{q}}^{\prime }$. So whenever *i*_*p*_ = *k*_*q*_, $\left\{a_{i_{p}}\in C_{j_{p}}\right\}=\left\{a_{k_{q}}^{\prime }\in C_{l_{q}}^{\prime }\right\}$.

(2) Since $R_{1}$ and $R_{2}$ are consequent bound PAAs, $con(R_{1})$ and $con(R_{2})$ are inconsistent. So *i*_*n*_ = *k*_*m*_ and $a_{i_{n}}=a_{k_{m}}^{\prime }$ but $C_{j_{n}}\cap C_{l_{m}}^{\prime }=⊥$. So *i*_*n*_ = *k*_*n*_ but $\left\{a_{i_{n}}\in C_{j_{n}}\right\}\neq \left\{a_{k_{m}}^{\prime }\in C_{l_{m}}^{\prime }\right\}$.

So $R_{1}$ and $R_{2}$ are consequent bound CPRs.

**Notation**. For a BKO *B*, *Abox*(*B*) represents *B*’s A-box. Similarly, *Tbox*(*B*) represents *B*’s T-box.

**Theorem 5.2.1**. Let *B* be a BKO. *Abox*(*B*) is a BKB.

*Proof*. Let *B* be a BKO and let *Abox*(*B*) be the set of all PAAs in *B*. We will show that (1) for any distinct PAAs $R_{1},R_{2}\in Abox\left(B\right)$ either $R_{1}$ is mutually exclusive with $R_{2}$ or $con\left(R_{1}\right)\neq con\left(R_{2}\right)$; and (2) for any subset *S* of mutually consequent bound CPRs of *B*, ∑_*Q*∈*S*_ *P*(*Q*) ≤ 1.

(1) Let $R_{1}$ and $R_{2}$ be distinct elements of *Abox*(*B*). Since $R_{1},R_{2}\in B$, either they are mutually exclusive or $con(R_{1})$ is consistent with the negation $con(R_{2})$ and $con(R_{2})$ is consistent with the negation of $con(R_{1})$. If the PAAs $R_{1}$ and $R_{2}$ are mutually exclusive PAAs, then by Lemma 5.2.1 they are mutually exclusive by CPRs. And if $con(R_{1})$ and the negation of $con(R_{2})$ are consistent (and vice versa), either *a*_1_ ≠ *a*_2_ or *C*_1_∩¬*C*_2_ ≠ ⊥ and ¬*C*_1_∩*C*_2_ ≠ ⊥. So either *a*_1_ ≠ *a*_2_ or *C*_1_ ≠ *C*_2_. So $con\left(R_{1}\right)\neq con\left(R_{2}\right)$.

(2) Since *B* is a BKO, for any subset *S* of mutually consequent bound PAAs of *B*, ∑_*Q*∈*S*_ *P*(*Q*) ≤ 1. And by Lemma 5.2.2, if $R_{1}$ and $R_{2}$ are consequent bound PAAs, they are consequent bound CPRs. So *S* remains unchanged and ∑_*Q*∈*S*_ *P*(*Q*) ≤ 1.

Note that an equivalent version of Theorem 5.2.1 appears in [11] as Lemma 7.1.

**Proposition 5.2.1**. (1) For a fully instantiated BKO *B*, any marginal or posterior probabilities which could be calculated using the pmf induced by *B* are identical to those calculated using the pmf induced by *Abox*(*B*). (2) Additionally, any marginal or posterior probabilities which are incalculable using the pmf induced by *Abox*(*B*) will also be incalculable using the pmf induced by *B*.

Having proven that a BKO has an equivalent BKB, we will turn our attention to the question of how to generate it.

### 5.3 A reasoning algorithm

The Full Instantiation Algorithm will fully instantiate a BKO. To achieve this, the algorithm begins with a set of PAAs, denoted *H*. This set is empty by default, but it is not required to be empty. First PAAs with empty antecedents are appended to *H*, followed by PAAs supported by H. Then, any combination of PTA and instantiation function that will result in a PAA supported by *H* is also added. This process is repeated until no additional PAAs are added to *H*.

**Definition 5.3.1**. The *generalization* of assertion *a* ∈ *C*, denoted *gen*(*a* ∈ *C*) is $\hat{x}\in \hat{C}$ where $\hat{x}$ is a variable individual and $\hat{C}$ is a variable concept, with each specific individual in *C* is replaced with a variable individual.

**Definition 5.3.2**. Two variable assertions ${\hat{x}}_{1}\in f_{1}\left(L\left(Q\right),\left\{{\hat{x}}_{1},...,{\hat{x}}_{w}\right\}\right)$ and ${\hat{y}}_{1}\in f_{2}\left(L\left(Q\right),\left\{{\hat{y}}_{1},...,{\hat{y}}_{w}\right\}\right)$ are *equivalent* if $f_{1}\left(L\left(Q\right),\left\{{\hat{z}}_{1},...,{\hat{z}}_{w}\right\}\right)=f_{2}\left(L\left(Q\right),\left\{{\hat{z}}_{1},...,{\hat{z}}_{w}\right\}\right)$ for any $\left\{{\hat{z}}_{1},...,{\hat{z}}_{w}\right\}$

**Definition 5.3.3**. An instantiation function *g* is *compatible* with PTA *T* if *g* is a one to one mapping from *I*(*T*) to a set of specific individuals.

The Full Instantiation Algorithm takes two arguments. The first is a BKO *B*. The second is an initial reasoning anchor *H*_*i*_, defaulting to the empty set. The Full Instantiation Algorithm returns a BKO.

**Proposition 5.3.1**. The output of the Full Instantiation Algorithm is a BKO

Note that this proposition follows from Theorem 5.1.1, which states that the union of a BKO *B* and the instantiation of any PTA in *B* is still a valid BKO.

### 5.4 Complexity of the algorithm

The Full Instantiation Algorithm’s complexity is driven by the instantiation of PTAs. Consider the general form of the PTA:

$$\begin{array}{c} T:P({\hat{x}}_{i_{n}}\in {\hat{C}}_{j_{n}}|{\hat{x}}_{i_{1}}\in {\hat{C}}_{j_{1}}\wedge ...\wedge {\hat{x}}_{i_{n-1}}\in {\hat{C}}_{j_{n-1}})=p \end{array}$$

For each variable assertion ${\hat{x}}_{i_{k}}\in {\hat{C}}_{j_{k}}$ in *ant*(*T*), where 1 ≤ *k* ≤ *n*−1, let |*M*_*k*_| represent the set of variable assertions that generalize to ${\hat{x}}_{i_{k}}\in {\hat{C}}_{j_{k}}$. Let *S*_*T*_ be the set of PAAs instantiated from PTA *T*. Then

$$\begin{array}{c} |S_{T}|\leq \prod_{k=1}^{n-1}\left|M_{k}\right| \end{array}$$

**Full Instantiation Algorithm**

1: Let *H*^−^ = *Null*, *H* = *H*_*i*_

2: **while** *H* ≠ *H*^−^ **do**

3:  *H* = *H*^−^

4:  H=H∪{R∈Abox(B)|S⊆HsupportsR}

5:  **for** *T*_*i*_ ∈ *Tbox*(*B*) **do**

6:   *G* = ∅

7:   D={con(R)|R∈H}

8:   **for** $T_{i_{j}}\in ant\left(T_{i}\right)$ **do**

9:    **for** *D*_*k*_ ∈ *D* **do**

10:     **if** $gen\left(D_{k}\right)=T_{i_{j}}$ **then**

11:      $g_{i_{j}}:I\left(ant\left(T_{i_{j}}\right)\right)\to I\left(D_{k}\right)$

12:      $G=G\cup \{g_{i_{j}}\}$

13:   **for** $\{g=\cup_{l=1}^{n}g_{l}|\left\{g_{1},...,g_{n}\right\}\subseteq G$,$\cup_{l=1}^{n}g_{l}$ is compatible with *T*_*i*_} **do**

14:  *H* = *H* ∪ *T*_*i*_|_*g*_

15: **return** *H*

The product $\prod_{k=1}^{n-1}\left|M_{k}\right|$ is an upper bound on |*S*_*T*_|. So the worst case time complexity is *O*(*M*^*n*^), where *M* largest number of variable assertions that are generalized to a PTA. The space complexity is also exponential, because the time complexity is driven by the number of new assertions being instantiated and is directly related to the size of the BKO. This will be true for both probabilistic and non-probabilistic assertions, because it depends on how many PAAs already in the BKO can be combined to instantiate new PAAs and not what their probability is. However, the case where $|S_{T}|=\prod_{k=1}^{n-1}\left|M_{k}\right|$ occurs when there are no shared variable individuals between variable assertions in *ant*(*T*). Consider the antecedent of *T*:

$$\begin{array}{c} ant\left(T\right)={\hat{x}}_{i_{1}}\in {\hat{C}}_{j_{1}}\wedge ...\wedge {\hat{x}}_{i_{n-1}}\in {\hat{C}}_{j_{n-1}} \end{array}$$

Assume for some variable assertions ${\hat{x}}_{i_{p}}\in {\hat{C}}_{j_{p}},{\hat{x}}_{i_{l}}\in {\hat{C}}_{j_{l}}$ there exists some ${\hat{x}}_{i_{q}}$ such that ${\hat{x}}_{i_{q}}$ is included in the variable concepts ${\hat{C}}_{j_{p}}$ and ${\hat{C}}_{j_{l}}$. Then the set of assertions that generalize to ${\hat{x}}_{i_{p}}\in {\hat{C}}_{j_{p}}$, denoted $M_{p}^{*}$, may include fewer assertions than the original *M*_*p*_. Similarly, we can denote $M_{l}^{*}$ as the set of assertions that generalize to ${\hat{x}}_{i_{l}}\in {\hat{C}}_{j_{l}}$. So the number of PAAs instantiated from the Full Instantiation Algorithm is

$$\begin{array}{c} |S_{T}|=\prod_{k=1}^{n-1}|M_{k}^{*}|\leq \prod_{k=1}^{n-1}\left|M_{k}\right| \end{array}$$

Although in this case |*S*_*T*_| is less than the upper bound, it still may grow exponentially with respect to the length of *T*’s antecedent. We illustrate this with an example. Consider the PTA:

$$\begin{array}{c} T:P({\hat{x}}_{1}\in R_{1}\left({\hat{x}}_{1},{\hat{x}}_{2}\right)|{\hat{x}}_{1}\in R_{2}\left({\hat{x}}_{1},{\hat{x}}_{3}\right),{\hat{x}}_{4}\in R_{3}\left({\hat{x}}_{4},{\hat{x}}_{5}\right),{\hat{x}}_{6}\in R_{4}\left({\hat{x}}_{6},{\hat{x}}_{7}\right))=p \end{array}$$

Note that there is no overlap between the members of *ant*(*T*), there are no variable individuals that are shared between variable assertions in *T*’s antecedent. Now assume for a given BKO, we have three PAAs whose generalization is ${\hat{x}}_{1}\in R_{2}\left({\hat{x}}_{1},{\hat{x}}_{3}\right)$, four PAAs whose generalization is ${\hat{x}}_{4}\in R_{3}\left({\hat{x}}_{4},{\hat{x}}_{5}\right)$, and three PAAs whose generalization is ${\hat{x}}_{6}\in R_{4}\left({\hat{x}}_{6},{\hat{x}}_{7}\right)$. Then we can infer thirty-six PAAs from *T*. Clearly, the number of times that a PTA may be instantiated is exponential with respect to the length of its antecedent. A similar problem can be seen regarding knowledge acquisition in Bayesian Networks. One advantage that BKO theory has, in addition to handling cycles and incompleteness, is that not all combinations of PTAs are possible. This is best communicated through an example. Consider the following PTA:

$$\begin{array}{c} T:P({\hat{x}}_{1}\in R_{1}\left({\hat{x}}_{1},{\hat{x}}_{2}\right)|{\hat{x}}_{1}\in R_{2}\left({\hat{x}}_{1},{\hat{x}}_{3}\right),{\hat{x}}_{4}\in R_{3}\left({\hat{x}}_{4},{\hat{x}}_{2}\right),{\hat{x}}_{3}\in R_{4}\left({\hat{x}}_{3},{\hat{x}}_{2}\right))=p \end{array}$$

There could be many PAAs whose consequents are generalizations of ${\hat{x}}_{3}\in R_{4}\left({\hat{x}}_{3},{\hat{x}}_{2}\right)$, but an instantiation function will only be valid if it maps ${\hat{x}}_{3}$ to the same specific individual as it does for ${\hat{x}}_{1}\in R_{2}\left({\hat{x}}_{1},{\hat{x}}_{3}\right)$. So if we have we have three PAAs that are generalizations of ${\hat{x}}_{1}\in R_{2}\left({\hat{x}}_{1},{\hat{x}}_{3}\right)$, four PAAs that are generalizations of ${\hat{x}}_{4}\in R_{3}\left({\hat{x}}_{4},{\hat{x}}_{2}\right)$, and three PAAs that are instantiations of ${\hat{x}}_{3}\in R_{4}\left({\hat{x}}_{3},{\hat{x}}_{2}\right)$. Then we cannot infer thirty-six PAAs as before. There will be some combinations that would require ${\hat{x}}_{3}$ to be mapped to multiple specific individuals by the same instantiation functions, which would not be valid. This can greatly reduce the number of PAAs that are instantiated.

There is one special case that represents many real-world applications and must be highlighted. Many ontologies, particularly in the biomedical domain, have terminological axioms that can be represented as PTAs of the form:

$$\begin{array}{c} T:P({\hat{x}}_{i_{2}}\in {\hat{C}}_{j_{2}}|{\hat{x}}_{i_{1}}\in {\hat{C}}_{j_{1}})=p \end{array}$$

In this case, the number of PAAs instantiated is equal to the number of assertions that generalize to ${\hat{x}}_{i_{1}}\in {\hat{C}}_{j_{1}}$ in the BKO.

### 5.5 Answering the probabilistic membership query

BKOs can be used to answer probabilistic membership queries (PMQs), thereby perform the probabilistic analogs of the standard DL reasoning tasks of instance and relation checking. This can be done for both fully instantiated BKOs as well as ones that are not yet fully instantiated. We rely on a BKB reasoning technique called *partial belief revision*.

Let *B* be a BKB. Let *Q* be a query of the form

$$\begin{array}{c} P(V_{j_{1}}=v_{j_{1}}\wedge ...\wedge V_{j_{m}}=V_{j_{m}}|V_{i_{1}}=v_{i_{1}}\wedge ...\wedge V_{i_{n}}=v_{i_{n}})=p \end{array}$$

with probability *p*, such that for all *V*_*x*_ = *v*_*x*_ ∈ *Q*, *V*_*x*_ = *v*_*x*_ ∈ *B*. We refer to *con*(*Q*) as the *reasoning target* and *ant*(*Q*) as the *evidence*. In order to solve this with BKB theory’s belief updating techniques, we must define a query rv *VQ* such that *r*(*VQ*) = {True, False}, and a query CPR RQ such that ant(RQ)=con(Q) and con(RQ)={VQ=True}. Let BQ=B∪RQ. Then *p* is computable as the belief updating problem *p* = *P*(*VQ* = True |*ant*(*Q*)). Intuitively, this process adds a CPR whose probability is equal to *p* and can be solved using belief updating.

BKOs can be used to solve PMQs in a similar way. Let *B* be a BKO, and let *Q* be a probabilistic membership query of the form

$$\begin{array}{c} P(V_{j_{1}}=\left\{a_{j_{1}}\in C_{j_{1}}\right\}\wedge ...\wedge V_{j_{m}}=\left\{a_{j_{m}}\in C_{j_{m}}\right\}|V_{i_{1}}=\left\{a_{i_{1}}\in C_{i_{1}}\right\}\wedge ...\wedge V_{i_{n}}=\left\{a_{i_{n}}\in C_{i_{n}}\right\}) \end{array}$$

with probability *p*, such that every clause *a*_*x*_ ∈ *C*_*x*_ is a consequent of at least one PAA in *B*. After *B* is fully instantiated, the PMQ can be solved using the same techniques just described for BKBs. This is because, as we have shown, a BKO’s A-box is a valid BKB.

Previously, we answered the PMQ by first fully instantiating the BKO to a BKB and then performing partial belief revision. Suppose we would like to set ungrounded belief conditions as evidence. To do so, let *B* be a BKO and *Q* be a probabilistic membership query of the form

$$\begin{array}{c} P(V_{j_{1}}=\left\{a_{j_{1}}\in C_{j_{1}}\right\}\wedge ...\wedge V_{j_{m}}=\left\{a_{j_{m}}\in C_{j_{m}}\right\}|V_{i_{1}}=\left\{a_{i_{1}}\in C_{i_{1}}\right\}\wedge ...\wedge V_{i_{n}}=\left\{a_{i_{n}}\in C_{i_{n}}\right\}) \end{array}$$

with *p* the probability to compute, such that every clause $a_{j_{x}}\in C_{j_{x}}$ is a consequent of at least one PAA in *B*. Note that unlike before, the members of the antecedent of *Q* do not have to be a consequent of a PAA in *B*. Now using *Q*’s antecedent, create a set *S* of PAAs {*S*_1_, …, *S*_*n*_} such that each *S*_*k*_ is the PAA $\text{P}\left(a_{i_{k}}\in C_{i_{k}}\right)=p_{k}$, where *p*_*k*_ is an unspecified probability. Using *S* as an input initial reasoning anchor, fully instantiate *B* using the Full Instantiation Algorithm. Then *p* can be computed using BKB theory’s partial relief revision. Since the members of *ant*(*Q*) are not necessarily all in *B*, the algorithm will build the fully instantiated BKO starting with the set *S*. In partial belief revision, these antecedent conditions are considered evidence, so the unspecified probabilities *p*_*k*_ will not contribute to the result.

## 6 Knowledge fusion with BKOs

Current methods for merging ontologies require knowledge to be rejected or altered to prevent contradicting information. This section introduces BKO fusion, where reasoning can occur regardless of whether or not contradictions are present. BKO fusion eliminates the need to check for inconsistencies and remedy them through manual or automated means. Not only is all knowledge from the input ontologies retained in the fused one, but new inferences, not present in the individual ontologies, are generated. This section begins with the theoretical framework of BKOs, followed by the BKO Fusion Algorithm, and lastly a discussion on the role of ontology alignment.

### 6.1 Theoretical framework

BKOs leverage their close relationship to BKBs to apply Bayesian Knowledge Fusion to the problems in ontology alignment that arise when there is uncertain knowledge. The concept and formulation are both analogous to BKB fusion. Conflicting knowledge from different sources is modeled as knowledge fragments with associated relative reliability weightings. This approach allows for Bayesian inferencing about conflicting information. Note that, because BKOs are a generalization of classical ontologies, these methods apply equally to BKOs and classical ontologies.

**Definition 6.1.1**. A *source class*, *C*_*s*_, is a class representing that knowledge came from a source *s*.

**Definition 6.1.2**. A *source assertion* *a* ∈ *C*_*s*_ is an assertion indicating membership in a source class.

**Definition 6.1.3**. A *source random variable* *V*_*s*_ is a random variable such that *r*(*V*_*s*_) is a set of source assertions.

**Definition 6.1.4**. For a PAA R and source random variable *V*_*s*_, R is referred to as a *sourced PAA* if $V_{s}\in ant\left(R\right)$. A PTA *T* is referred to as a *sourced PTA* if source assertion {*a* ∈ *C*_*s*_}∈*ant*(*T*)

**Definition 6.1.5**. A *BKO Fragment* is a triple (*B*, *s*, *w*) where *B* is a BKO, *s* is a term representing the source of the knowledge contained in *B*, and *w* > 0 is a real number representing the reliability of *s* in comparison to other sources.

Note that a single ontology can be represented by multiple BKO Fragments. Different sources can have different reliabilities on different subsets of their domain of discourse, and those subsets are represented as fragments. A source might provide multiple fragments to a fused model, each with a different reliability weighting.

The BKO Fusion Algorithm takes two arguments. The first is a set of BKO Fragments **F** = {*F*_1_ = (*B*_1_, *s*_1_, *w*_1_), …, *F*_*n*_ = (*B*_*n*_, *s*_*n*_, *w*_*n*_)} such that for any fragments *F*_*i*_, *F*_*j*_ ∈ **F**, *s*_*i*_ ≠ *s*_*j*_. The second argument is an initial reasoning anchor *H*_*i*_, defaulting to the empty set. To model the source that a PAA or PTA from a BKO Fragment *F* came from, we include a source random variable in the antecedent of each PAA and a source assertion in the antecedent of each PTA.

**BKO Fusion Algorithm**

1: *w* = 0

2: **for** *F*_*i*_ ∈ **F** **do**

3:  *w* = *w* + *w*_*i*_

4: **for** *F*_*i*_ ∈ **F** **do**

5:  $R_{s_{i}}:P\left(V_{s_{i}}=\left\{a_{s_{i}}\in C_{s_{i}}\right\}\right)=\frac{wi}{w}$

6:  $B_{i}=B_{i}\cup R_{s_{i}}$

7:  **for** all PAAs $R_{i_{j}}\in B_{i}$ **do**

8:   $ant\left(R_{i_{j}}\right)=ant\left(R_{i_{j}}\right)\wedge \left\{V_{s_{i}}=a_{s_{i}}\in C_{s_{i}}\right\}$

9:  **for** all PTAs *T*_*i*_ ∈ *B*_*i*_ **do**

10:   $ant\left(T_{i_{j}}\right)=ant\left(T_{i_{j}}\right)\wedge a_{s_{i}}\in C_{s_{i}}$

11: $B=\cup_{i=1}^{n}B_{i}$

12: **return** *B*

The result of BKO Fusion is a valid BKO, as we will show in the following theorem. The proof depends on a crucial assumption. The definition of a BKO depends on knowing whether classes are disjoint or not. If that information about classes from different ontologies is not known, we must assume that there are no classes *C*_*i*_ ∈ *C*(*B*_*i*_) and *C*_*j*_ ∈ *C*(*B*_*j*_) such that *C*_*i*_∩*C*_*j*_ = ⊥. Similarly, we also must assume that no classes such that *C*_*i*_ ∩ ¬*C*_*j*_ = ⊥ or *C*_*j*_ ∩ ¬*C*_*i*_ = ⊥ unless that information is provided. Such information would be included in an alignment ontology, which can be included as an input to the fusion algorithm.

**Theorem 6.1.1**. For any two BKO fragments *F*_*i*_, *F*_*j*_ ∈ **F** such that *s*_*i*_ ≠ *s*_*j*_, the result of BKO Fusion will be a valid BKO.

*Proof*. Let *F*_*i*_ = (*B*_*i*_, *s*_*i*_, *w*_*i*_) and *F*_*j*_ = (*B*_*j*_, *s*_*j*_, *w*_*j*_) such that *s*_*i*_ ≠ *s*_*j*_ and let $B=B_{i}\cup B_{j}\cup \left\{R_{s_{i}}\right\}\cup \left\{R_{s_{j}}\right\}$. Since *B*_*i*_ and *B*_*j*_ are a set of PAAs and PTAs, and $R_{s_{i}}$ and $R_{s_{j}}$ are themselves PAAs, *B* is a set of PAAs and PTAs. Now, we show that it sets the four conditions set in Definition 4.3.6:

i Let $R_{i}=\left\{R_{i_{1}},\ldots ,R_{i_{n}}\right\}$ and $R_{j}=\left\{R_{j_{1}},\ldots ,R_{j_{m}}\right\}$ be the set of PAAs in *B*_*i*_ and *B*_*j*_, respectively. Since we assume all classes *C*_*i*_ and *C*_*j*_ are disjoint, for any $R_{i_{k}}\in R_{i}$ and $R_{j_{l}}\in R_{j}$, we can say that $con(R_{i_{k}})$ is consistent with the negation of $con(R_{j_{l}})$ and $con(R_{j_{l}})$ is consistent with the negation of $con(R_{i_{k}})$. Additionally, source PAAs $R_{s_{i}}$ and $R_{s_{j}}$ have different individuals in their consequent that are unique to each source PAA. So (1) $con(R_{s_{i}})$ is consistent with the negation of $con(R_{s_{j}})$ and $con(R_{s_{j}})$ is consistent with the negation of $con(R_{s_{i}})$, and (2) for any $R_{m}\in R_{i}\cup R_{j}$, both $con(R_{s_{i}})$ and $con(R_{s_{j}})$ are consistent with the negation of $con(R_{m})$ and $con(R_{m})$ is consistent with the negation of both $con(R_{s_{i}})$ and $con(R_{s_{j}})$. So, for any $R_{1},R_{2}\in B$, either $R_{1}$ is mutually exclusive with $R_{2}$ or $con(R_{1})$ is consistent with the negation of $con(R_{2})$ and $con(R_{2})$ is consistent with the negation of $con(R_{1})$.
ii Let $T_{i}=\left\{T_{i_{1}},\ldots ,T_{i_{n}}\right\}$ and $T_{j}=\left\{T_{j_{1}},\ldots ,T_{j_{m}}\right\}$ be the set of PTAs in *B*_*i*_ and *B*_*j*_, respectively. Each member of *T*_*i*_ has the source assertion $a_{s_{i}}\in C_{s_{i}}$ in its antecedent. Similarly, every member of *T*_*j*_ has the source assertion $a_{s_{j}}\in C_{s_{j}}$ in its antecedent. Since the source assertions are not variable assertions, for any instantiation functions *g*_1_, *g*_2_, and any $T_{i_{k}}\in T_{i}$ and $T_{j_{l}}\in T_{j}$, the source random variables of $T_{i_{k}}|g_{i}$ and $T_{j_{l}}|g_{j}$ will be $V_{s}=\left\{a_{s}\in C_{s_{i}}\right\}$ and $V_{s}=\left\{a_{s}\in C_{s_{j}}\right\}$, respectively. And since classes from different ontologies are not disjoint, for any $T_{i_{k}}\in B_{i}$ and $T_{j_{l}}\in B_{j}$, $con(T_{i_{k}}|g_{i})$ is consistent with the negation of $con(T_{j_{l}}|g_{j})$ and $con(T_{j_{l}}|g_{j})$ is consistent with the negation of $con(T_{i_{k}}|g_{i})$.
iii Let $R_{i}=\left\{R_{i_{1}},\ldots ,R_{i_{n}}\right\}$ be the set of PAAs in *B*_*i*_ and $T_{i}=\left\{T_{i_{1}},\ldots ,T_{i_{m}}\right\}$ be the set of PTAs in *B*_*i*_. Also, let $R_{j}=\left\{R_{j_{1}},\ldots ,R_{j_{k}}\right\}$ be the set of PAAs in *B*_*j*_ and $T_{j}=\left\{T_{j_{1}},\ldots ,T_{j_{l}}\right\}$ be the set of PTAs in *B*_*j*_. The BKO Fusion Algorithm appends a source random variable $V_{s_{i}}=\left\{a_{s_{i}}\in C_{s_{i}}\right\}$ to each member of $R_{i}$ and a source assertion $a_{s_{i}}\in C_{s_{i}}$ to each member of *T*_*i*_. Similarly, the BKO Fusion Algorithm appends a source random variable $V_{s_{j}}=\left\{a_{s_{j}}\in C_{s_{j}}\right\}$ to each member of $R_{j}$ and a source assertion $a_{s_{j}}\in C_{s_{j}}$ to each member of *T*_*j*_. Because the source assertions are not variable assertions, they do not change between instantiation functions. And since no classes are disjoint across different BKOs, for any PTA or PAA $Q_{i}\in R_{i}\cup T_{i}$ and PTA or PAA $Q_{j}\in R_{j}\cup T_{j}$, *con*(*Q*_*i*_) will be consistent with the negation of *con*(*Q*_*j*_) and *con*(*Q*_*j*_) will be consistent with the negation of *con*(*Q*_*i*_). And since source assertions are consistent with the negation of any assertion in *B*_*i*_∪*B*_*j*_, for any PAA R and PTA *T* in BKO *B*, and for any instantiation function *g*, either R and *T*|_*g*_ are mutually exclusive or con(R) is consistent with the negation of *con*(*T*|*g*) and *con*(*T*|*g*) is consistent with the negation of con(R).
iv Let *S* be a set of mutually consequent bound members of *B*. Then *S* cannot contain members from both *B*_*i*_ and *B*_*j*_, since, as shown before, the consequents of members of *B*_*i*_ and *B*_*j*_ are consistent. It also does not contain $R_{s_{i}}$ or $R_{s_{j}}$ with any members of *B*_*i*_ or *B*_*j*_ since the source assertions in the consequent of $R_{s_{i}}$ and $R_{s_{j}}$ cannot be inconsistent with any consequents in *B*_*i*_ or *B*_*j*_. So either *S* ⊆ *B*_*i*_, *S* ⊆ *B*_*j*_, or $S\subseteq \{R_{s_{i}},R_{s_{j}}\}$. *B*_*i*_ and *B*_*j*_ are valid BKOs, and we normalize the weights of $R_{s_{i}}$ and $R_{s_{j}}$, so for all sets *S* of mutually consequent bound members of *B*, ∑_*Q*∈*S*_ *P*(*Q*) ≤ 1

So for any two BKO fragments *F*_*i*_, *F*_*j*_ ∈ **F** such that *s*_*i*_ ≠ *s*_*j*_, the result of fusion by the BKO Fusion Algorithm will be a valid BKO.

Since the BKO returned from this algorithm is valid, it can be used as input to the Full Instantiation Algorithm. Then, all previously established BKB reasoning techniques can be applied to it. Therefore, as described in the previous section, the fused BKO can be used to answer probabilistic membership queries. Once the BKO is fused and fully instantiated, the process is identical to the one described in the previous section.

### 6.2 Complexity of BKO fusion

Let **F** = {*F*_1_, …, *F*_*n*_} be a set of BKO fragments. For some *F*_*i*_ ∈ **F**, we can write

$$\begin{array}{c} F_{i}=R_{i}\cup T_{i} \end{array}$$

Where $R_{i}$ and *T*_*i*_ are the set if PAAs and PTAs in *F*_*i*_, respectively. For each BKO being fused, the algorithm iterates over the set of PAAs and PTAs, which is equal to the size of each BKO Fragment:

$$\begin{array}{c} \sum_{i=1}^{n}|R_{i}\left|+\right|T_{i}|=\sum_{i=1}^{n}\left|F_{i}\right| \end{array}$$

So the complexity of the algorithm is *O*(*nm*) where *n* is the number of BKOs being fused and *m* is the number of PTAs and PAAs in the largest BKO Fragment. This is much faster than the Full Instantiation Algorithm. Although it may be necessary to run the two consecutively, first the BKO Fusion Algorithm then the Full Instantiation Algorithm, this is not always required. The Full Instantiation Algorithm is only required for reasoning over it as a BKB. Other applications of the fused ontologies can avoid that time consuming step.

It is important to note that it is not always necessary to fuse entire BKOs at one time. Often, only subsets of certain BKOs are of interest. In this case applying BKO Fusion to BKO subsets is preferred to save time.

### 6.3 BKO fusion and ontology alignment

When ontologies use different interpretations, their lexica must be related through some sort of mapping. This generally takes the form of an ontology dedicated to the purpose, a bridge ontology. (see [41] for recent work on this subject.) Ontology alignment has a strong need for an uncertainty formalism, because ontology interpretations are often vague, uncertain, and contentious. Even when the name of a class from one ontology is exactly the class name from another ontology, equating the two may still be incorrect if the classes are distinct or overlapping. A formal alignment ontology is necessary to avoid such issues. Ontology alignment methods exist, but are often deterministic and require that ultimately fiat decisions be made by humans or an algorithm. BKO theory is well-suited to alleviating this difficulty. It does not address the question of how to generate mappings, but it will model mappings containing uncertainty. Through fusion it permits the use of multiple dissonant mappings, each of which may themselves contain uncertainty. In such situations, formulate the ontologies to be aligned and the proposed mapping(s) each as individual BKO fragments and apply the algorithm to all the ontologies being fused and all the alignment ontologies. Every mapping used may contribute to the solution and offer up its insights.

This approach also simplifies the “meta-matching problem” of how to select a method for generating and evaluating mappings (see [42] for an example of recent work on this problem). Rather than being forced to select just one alignment strategy, many strategies may be selected simultaneously and their resultant mappings fused. This eases design requirements for automated alignment generators—they no longer need to eliminate or overrule uncertainty in a candidate alignment. Conflicting results become acceptable and even desirable if they accurately reflect real-world uncertainty and disagreement.

## 7 A detailed example

With an increase in the amount of data produced in the biological sciences there has also been an increase in the use of biological ontologies, such as Gene Ontology [43, 44], Human Phenotype Ontology [45], and the Infectious Disease Ontology [46]. They have applications in many areas of biomedicine [47] such as data integration [48, 49] and identifying protein-protein interactions [50, 51]. One problem that many biological researchers face is that although there are many available ontologies related to their domain, no single onotlogy adequately supports their research aims. As a result, many overlapping ontologies were developed to suit specific domains [52]. For example, the Human Disease Ontology (DO) [53] covers many human diseases. However, researchers studying epilepsy needed a more detailed ontology and created the Epilepsy Ontology [54]. BKO fusion can be applied to take information from separate ontologies and combine them into one. When sufficient information is available but spread out across different sources, creating an entirely new ontology in no longer necessary. This section presents a detailed example of the BKO fusion process, designed to highlight some of the unique and powerful characteristics of BKOs. We will show both how BKOs can be reasoned over despite contradictions and how new inferences can be formed as a result of fusion.

We fuse subsets of two ontologies, the Mondo Disease Ontology (MONDO) [55] and DO [53]. They cover a similar domain and are both OBO Foundry [56] ontologies, but fusing them is not trivial. These are not probabilistic ontologies but can be modeled as such by assigning each statement a probability of one. Our example will be centered around the sciatic nerve, the largest nerve in the body that runs from the lower back to the lower legs. The sciatic model is a popular model for studying nerve injury, due at least in part to its accessibility during surgery [57]. Although we can model any relation in either of these ontologies, we only use the “is a” relation in this example for clarity. Note that each class has a unique identifier, but we will instead use the common names to make the example easier to follow. If we need to specify which ontology the class comes from, we will add the ontology name in parentheses after the class name. For reference, Table 1 displays the common terms with their unique identifiers. We will start with the PTAs from each ontology and the bridge ontology between them. Then we will fuse them together, and finally we will reason over the resulting BKB.

**Table 1:** MONDO and DO identifiers and their common names.

Identifier	Common Name
MONDO:0006960	Sciatic Neuropathy
MONDO:0001543	Lesion of Sciatic Nerve
MONDO:0001397	Mononeuropathy
MONDO:0002121	Mononeuritis Simplex
MONDO:0002122	Neuritis
MONDO:0021166	Inflammatory Disease
DOID:114466	Sciatic Neuropathy
DOID:12528	Lesion of Sciatic Nerve
DOID:9473	Mononeuritis of Lower Limb
DOID:1188	Mononeuropathy
DOID:1802	Mononeuritis

### 7.1 Fusing two BKO Fragments

The following PTAs form a subset of MONDO:

$T_{M_{1}}:P\left(\hat{x}\in \text{Sciatic}\;\text{Neuropathy}|\hat{x}\in \;\text{Lesion}\;\text{of}\;\text{Sciatic}\;\text{Nerve}\right)=1$

$T_{M_{2}}:P\left(\hat{x}\in \text{Mononeuropathy}|\hat{x}\in \text{Sciatic}\;\text{Neuropathy}\right)=1$

$T_{M_{3}}:P\left(\hat{x}\in \text{Mononeuropathy}|\hat{x}\in \text{Mononeuritis}\;\text{Simplex}\right)=1$

$T_{M_{4}}:P\left(\hat{x}\in \text{Neuritis}|\hat{x}\in \text{Mononeuritis}\;\text{Simplex}\right)=1$

$T_{M_{4}}:P\left(\hat{x}\in \text{Inflammatory}\;\text{Disease}|\hat{x}\in \text{Mononeuritis}\right)=1$

The following PTAs form a subset of DO:

$T_{D_{1}}:P\left(\hat{x}\in \text{Lesion}\;\text{of}\;\text{Sciatic}\;\text{Nerve}|\hat{x}\in \text{Sciatic}\;\text{Neuropathy}\right)=1$

$T_{D_{2}}:P\left(\hat{x}\in \text{Mononeuritis}\;\text{of}\;\text{Lower}\;\text{Limb}|\hat{x}\in \text{Lesion}\;\text{of}\;\text{Sciatic}\;\text{Nerve}\right)=1$

$T_{D_{3}}:P\left(\hat{x}\in \text{Mononeuritis}|\hat{x}\in \text{Mononeuritis}\;\text{of}\;\text{Lower}\;\text{Limb}\right)=1$

$T_{D_{4}}:P\left(\hat{x}\in \text{Mononeuropathy}|\hat{x}\in \text{Mononeuritis}\right)=1$

The bridge ontology linking the terms from MONDO and DO was gathered from the EMBL-EBI Ontology xRef service (OxO) [58]. The following PTAs are from OxO:

$T_{B_{1}}:P\left(\hat{x}\in \text{Lesion}\;\text{of}\;\text{Sciatic}\;\text{Nerve}\;\text{(MONDO)}|\hat{x}\in \text{Lesion}\;\text{of}\;\text{Sciatic}\;\text{Nerve}\;\text{(DO)}\right)=1$

$T_{B_{2}}:P\left(\hat{x}\in \text{Lesion}\;\text{of}\;\text{Sciatic}\;\text{Nerve}\;\text{(DO)}|\hat{x}\in \text{Lesion}\;\text{of}\;\text{Sciatic}\;\text{Nerve}\;\text{(MONDO)}\right)=1$

$T_{B_{3}}:P\left(\hat{x}\in \text{Sciatic}\;\text{Neuropathy}\;\text{(MONDO)}|\hat{x}\in \text{Sciatic}\;\text{Neuropathy}\;\text{(DO)}\right)=1$

$T_{B_{4}}:P\left(\hat{x}\in \text{Sciatic}\;\text{Neuropathy}\;\text{(DO)}|\hat{x}\in \text{Sciatic}\;\text{Neuropathy}\;\text{(MONDO)}\right)=1$

$T_{B_{5}}:P\left(\hat{x}\in \text{Mononeuropathy}\;\text{(MONDO)}|\hat{x}\in \text{Mononeuropathy}\;\text{(DO)}\right)=1$

$T_{B_{6}}:P\left(\hat{x}\in \text{Mononeuropathy}\;\text{(DO)}|\hat{x}\in \text{Mononeuropathy}\;\text{(MONDO)}\right)=1$

$T_{B_{7}}:P\left(\hat{x}\in \text{Mononeuritis}\;\text{Simplex}\;\text{(MONDO)}|\hat{x}\in \text{Mononeuritis}\;\text{(DO)}\right)=1$

$T_{B_{8}}:P\left(\hat{x}\in \text{Mononeuritis}\;\text{(DO)}|\hat{x}\in \text{Mononeuritis}\;\text{Simplex}\;\text{(MONDO)}\right)=1$

**Fig 6:**
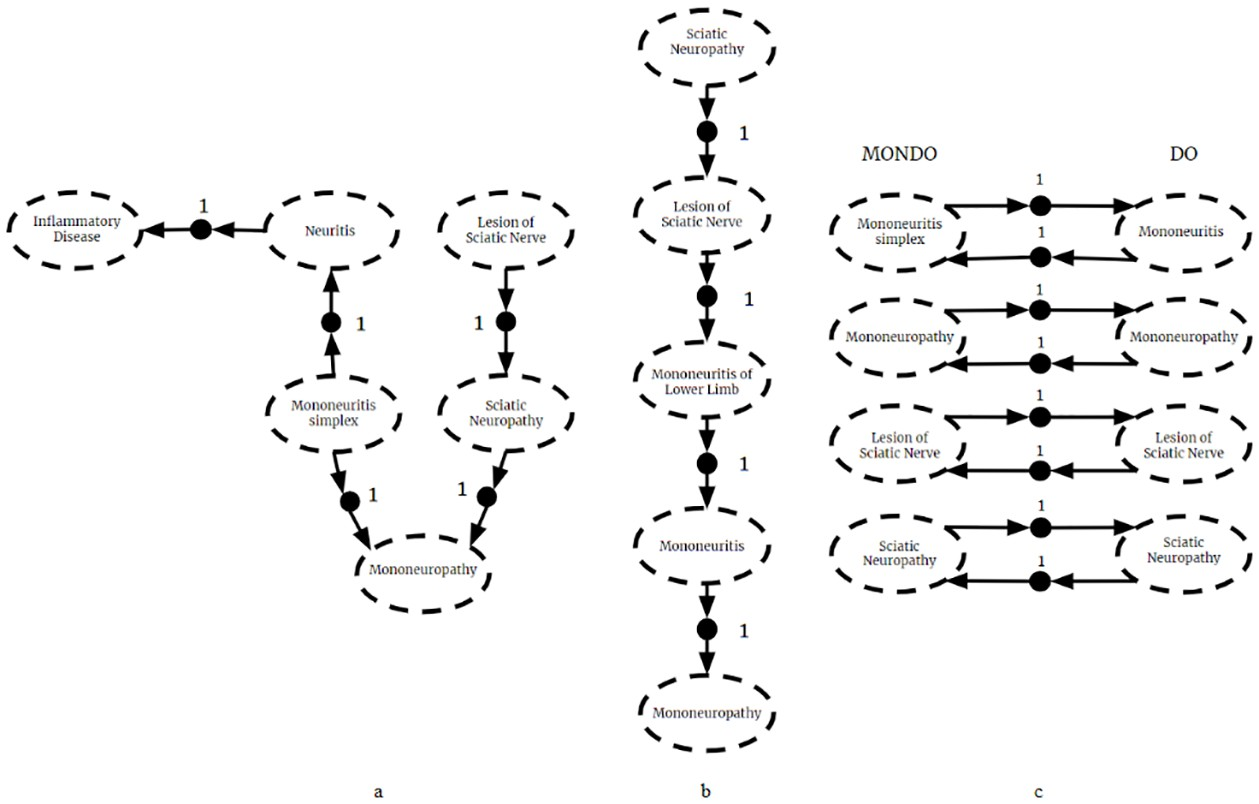
BKO fragments. Three BKO Fragments from (a) MONDO, (b) DO, and (c) the bridge from OxO.

These can be visualized in Fig 6. We follow the graph model for BKBs that was described in Section 3.4. Recall that the black nodes, called “S-nodes”, represent conditional probabilities. The other nodes, called “I-nodes”, represent random variable instantiations. The conditional probability being modeled in some S-node, *q*, is the probability of the I-node *q* points to given the I-node(s) that point to *q*.

**Fig 7:**
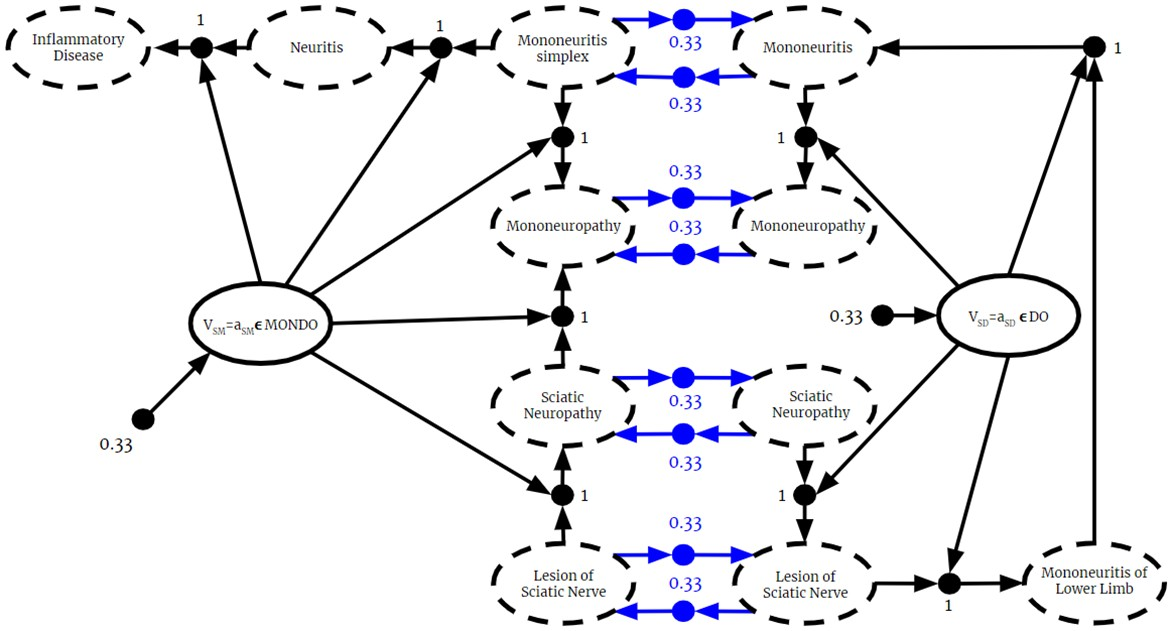
Combined BKO. The three BKO framgents combined to the same graph. At this stage the fusion algorithm is not yet complete because the BKO has not been fully instantiated. The dotted nodes represent terminological knowledge.

**Fig 8:**
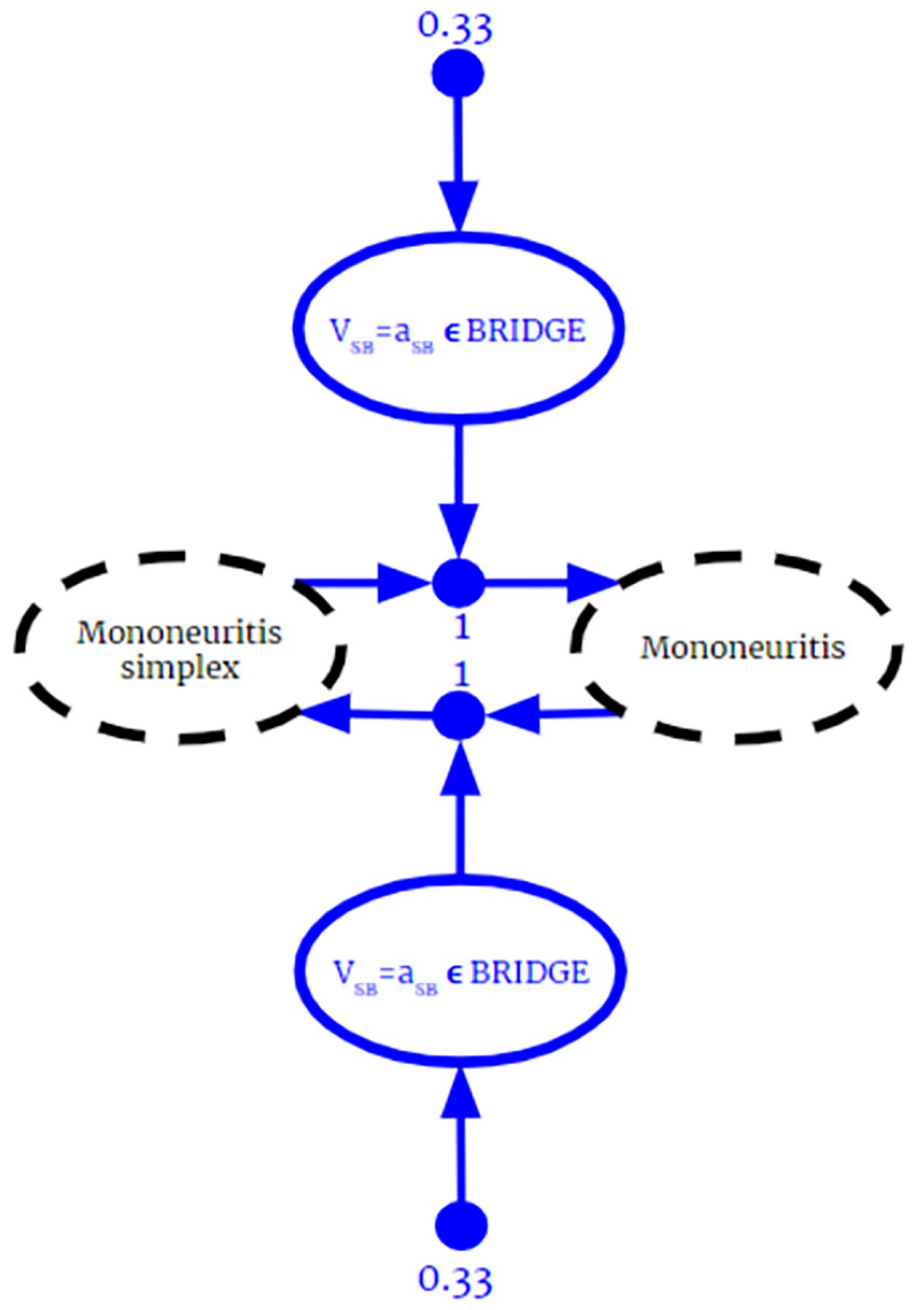
Expanded bridge nodes. The other figures in this section use a compressed representation of the nodes and edges that came from the bridge ontology. The blue nodes and edges in this example show what was compressed.

Based on the figure, it looks as though “Lesion of Sciatic Nerve” has no antecedent in MONDO and “Sciatic Neuropathy” has no antecedent in DO. This is not the case as we are only displaying a subset of each ontology. We can still start reasoning without including more information from MONDO or DO by using an initial reasoning anchor. We let

$$\left\{V_{a_{1}}=a\in \text{Lesion}\;\text{of}\;\text{Sciatic}\;\text{Nerve}\;\text{(MONDO)},V_{a_{2}}=a\in \text{Sciatic}\;\text{Neuropathy}\;\text{(DO)}\right\}$$

be our initial reasoning anchor using some individual *a* and consider three BKO Fragments: *F*_*M*_ : (*B*_*M*_, MONDO, 1), *F*_*D*_ : (*B*_*D*_, DO, 1), *F*_*B*_ : (*B*_*B*_, BRIDGE, 1). Here, we chose to set each weight to be 1. Since the algorithm normalizes the weights, their values only matter relative to each other, we could have set each weight to 2 and gotten the same result. They do not need to be equal either, but for this example we chose that they would be equal. Additionally, although not displayed in this example, multiple fragments from the same ontology could be included with different weights if desired. The fusion algorithm first adds source PAAs to the BKO and source random variables to the antecedents of each PAA or PTA in the input fragments. Graphically, this is shown in Fig 7. Here and in the remaining figures, we represent a compressed version the edges and nodes that come from the bridge ontology in blue. This is only for clarity, an example of what these blue nodes and edges represent is shown in Fig 8

This BKO is used as an input to the Full Instantiation Algorithm. At first sight, perhaps the most noticeable aspect of the BKB is the presence of cycles. However, BKBs are uniquely equipped to handle these cycles. With a closer look, one will notice a contradiction in as well. According to MONDO, a “Lesion of Sciatic Nerve” is a “Sciatic Neuropathy”. But according to DO, “Sciatic Neuropathy” is a “Lesion of Sciatic Nerve”. In many ontology merging approaches either MONDO or DO would need to be prioritized in this situation, and the other’s knowledge discarded. With BKOs, all knowledge from MONDO and DO can be included and reasoned about. But before we show that reasoning, we complete fusion by fully instantiating the BKO.

After starting with our initial reasoning anchor, we can instantiate two PAAs:

$$\begin{array}{c} R_{M_{1}}:P(V_{M_{1}}=\left\{a\in \text{Sciatic}\;\text{Neuropathy}\;\text{(MONDO)}\right\}|V_{M_{0}}=\{a\in \\ \text{Lesion}\;\text{of}\;\text{Sciatic}\;\text{Nerve}\;\text{(MONDO)}\}\wedge V_{s}=\left\{a_{s}\in \text{MONDO}\right\})=1 \end{array}$$

$$\begin{array}{c} R_{D_{1}}:P(V_{D_{1}}=\{a\in \text{Lesion}\;\text{of}\\ \text{Sciatic}\;\text{Nerve}\;\text{(DO)}\}|V_{D_{0}}=\left\{a\in \text{Sciatic}\;\text{Neuropathy}\;\text{(DO)}\right\}\wedge V_{s}=\left\{a_{s}\in \text{DO}\right\})=1 \end{array}$$

**Fig 9:**
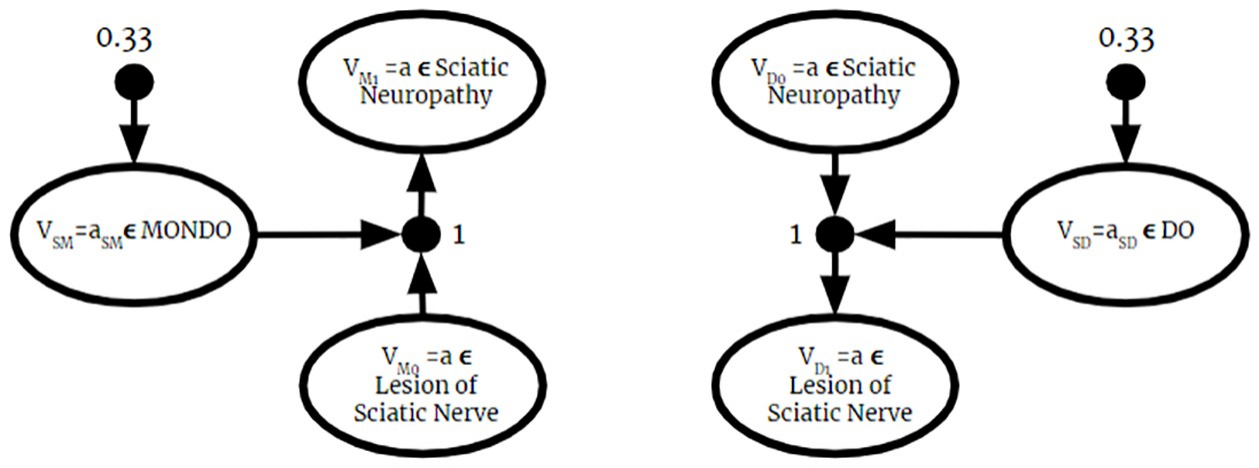
Instantiation first pass. From the initial reasoning anchor we can immediately instantiate two PTAs to PAAs.

Graphically, this is shown in Fig 9

At this point, *H* includes our initial reasoning anchor, $R_{M_{1}}$, and $R_{D_{1}}$. The following pass through the algorithm instantiates six more PTAs:

$R_{M_{2}}:P\left(V_{M_{2}}=a\in \text{Mononeuropathy}|V_{M_{1}}=a\in \text{Sciatic}\;\text{Neuropathy}\wedge V_{s}=\left\{a_{s}\in \text{MONDO}\right\}\right)=1$

$R_{D_{2}}:P\left(V_{D_{2}}=a\in \text{Mononeuritis}\;\text{of}\;\text{Lower}\;\text{Limb}|V_{D_{1}}=a\in \text{Lesion}\;\text{of}\;\text{Sciatic}\;\text{Nerve}\wedge V_{s}=\left\{a_{s}\in \text{DO}\right\}\right)=1$

$R_{B_{1}}:P\left(V_{M_{0}}=\left\{a\in \;\text{Lesion}\;\text{of}\;\text{Sciatic}\;\text{Nerve}\;\text{(MONDO)}\right\}|V_{D_{1}}=\left\{a\in \text{Lesion}\;\text{of}\;\text{Sciatic}\;\text{Nerve}\;\text{(DO)}\right\}\wedge V_{s}=\left\{a_{s}\in \text{BRIDGE}\right\}\right)=1$

$R_{B_{2}}:P\left(V_{D_{1}}=a\in \text{Lesion}\;\text{of}\;\text{Sciatic}\;\text{Nerve}\right|V_{M_{0}}=\left\{a\in \;\text{Lesion}\;\text{of}\;\text{Sciatic}\;\text{Nerve}\;\text{(MONDO)}\wedge V_{s}=\left\{a_{s}\in \text{BRIDGE}\right\}\right)=1$

$R_{B_{3}}:P(V_{M_{1}}=\left\{a\in \text{Sciatic}\;\text{Neuropathy}\;\text{(MONDO)}\right|V_{D_{0}}=\left\{a\in \text{Sciatic}\;\text{Neuropathy}\;\text{(DO)}\wedge V_{s}=\left\{a_{s}\in \text{BRIDGE}\right\}\right)=1$

$R_{B_{4}}:P(V_{D_{0}}=\left\{a\in \text{Sciatic}\;\text{Neuropathy}\;\text{(DO)}\right|V_{M_{1}}=\left\{a\in \text{Sciatic}\;\text{Neuropathy}\;\text{(MONDO)}\wedge V_{s}=\left\{a_{s}\in \text{BRIDGE}\right\}\right)=1$

**Fig 10:**
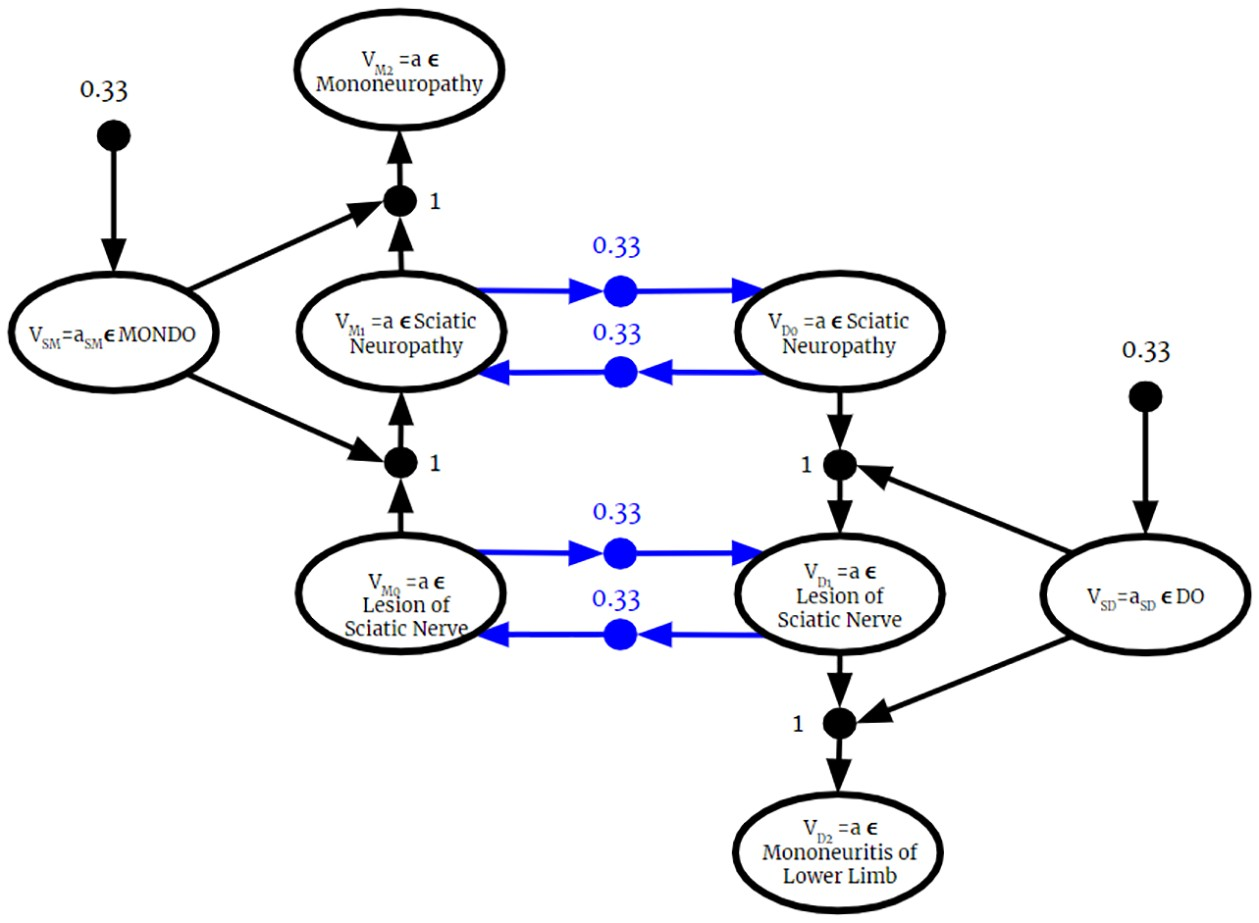
Instantiation second pass. A second pass through the Full Instantiation Algorithm increases the amount of PAAs in the BKO.

Or graphically as shown in Fig 10

**Fig 11:**
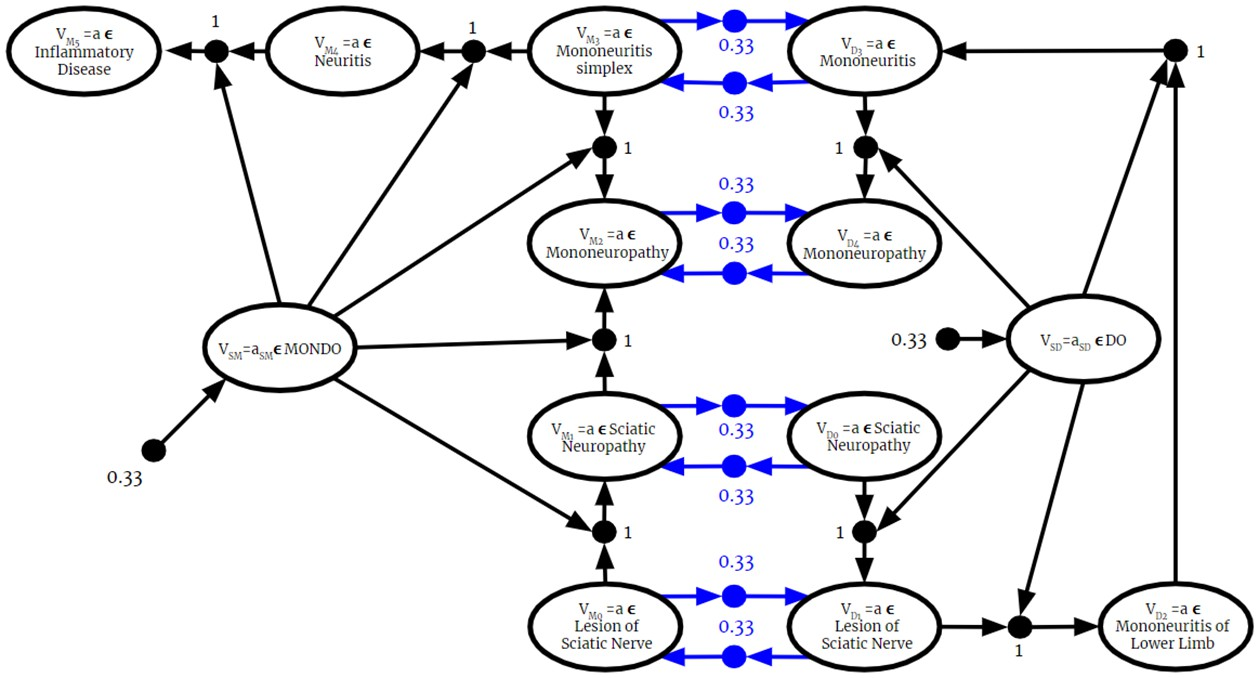
Fused BKO. The fused BKO that is returned by the BKO Fusion Algorithm.

A visualization of the BKB that the BKO Fusion algorithm returns is shown in Fig 11.

This is both a BKO and a BKB. Should the PTAs be returned along with a set of PAAs, in would no longer be a BKB but exclusively a BKO. But in order to make use of BKB reasoning we need the output to be a BKB.

### 7.2 BKO reasoning

**Fig 12:**
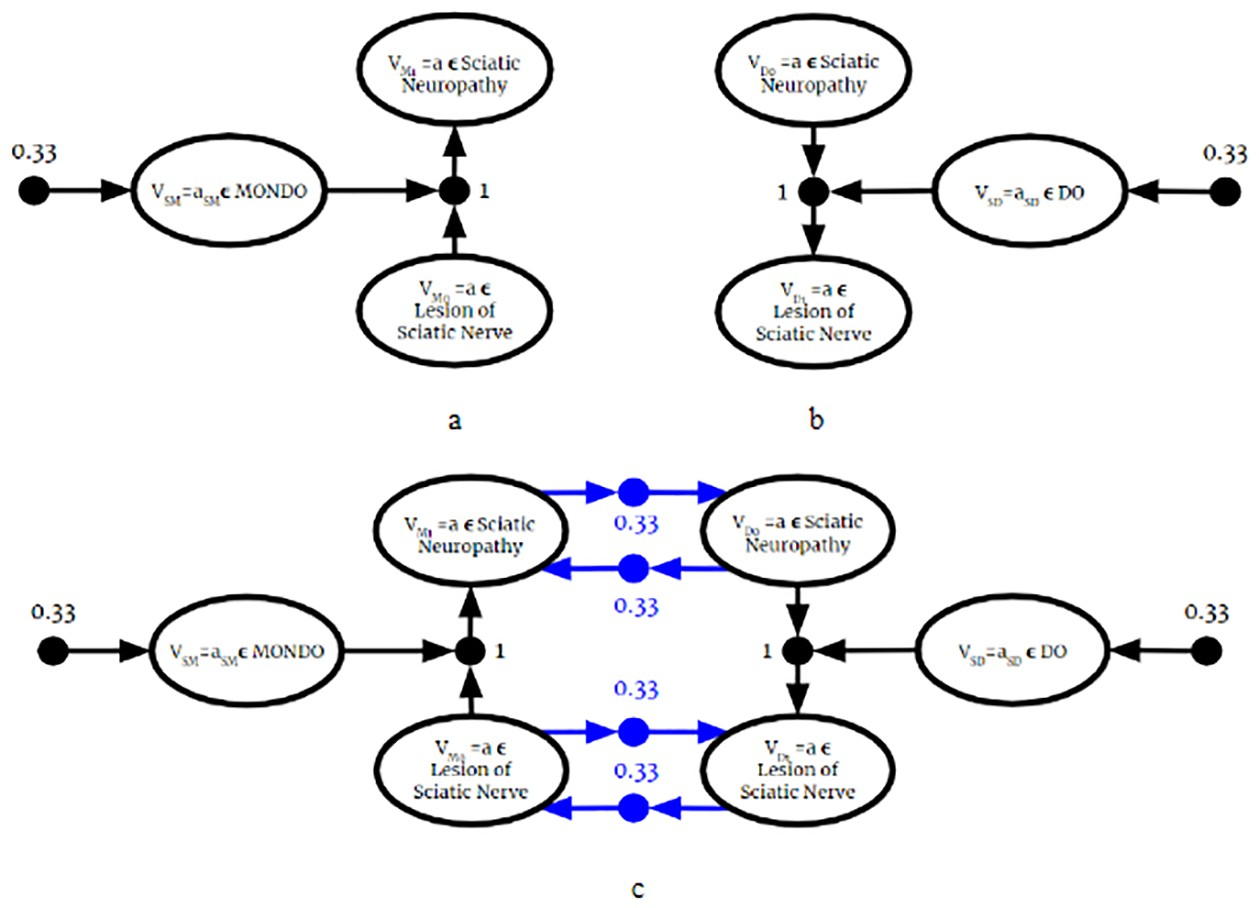
Handling contradictions. The two BKO fragments contradict each other. This contradiction does not present a problem when reasoning about a fused BKO. We can build two inferences, (a) from MONDO and (b) from DO from the larger BKO fragment (c).

Recall the definition of an *inference* over a BKB. There are many such inferences in our example, we will only focus on a few. However, one could consider all of them. This would result in a list of inferences with the probability of each inference allowing for comparison between them. When there is a contradiction within the ontology, this ranking can be used to determine which, if any, is more probable. Consider the subset of the BKB in Fig 12:

The probability of each inference is the product of the S-nodes in it. So here, *P*(*a*) = *P*(*b*) = 0.33. This result should be expected because we assign the same weight to each source. If we trusted one source more than another, that would be reflected in their final probability values. Rather than taking one assertion and discarding the other, we handle contradictions by returning both assertions with information on which one is more probable.

**Fig 13:**
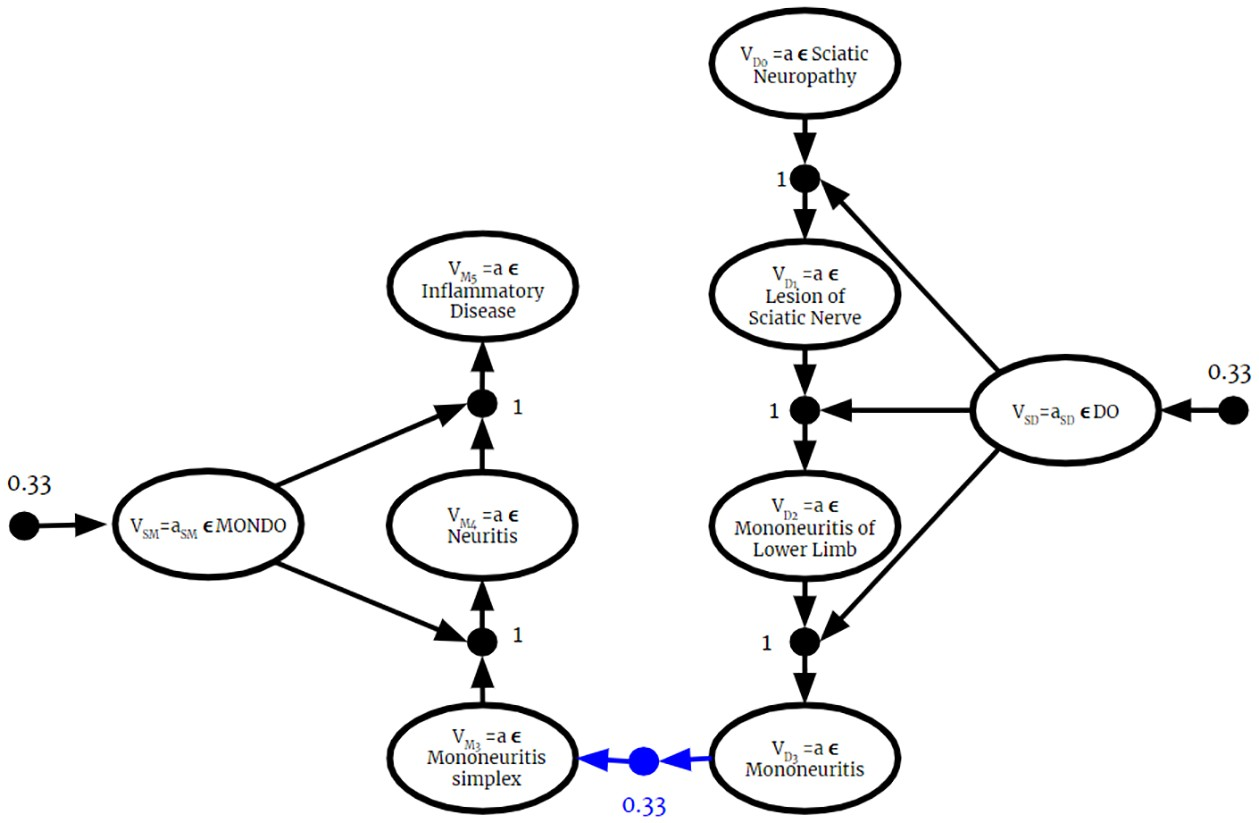
New inference. A new inference is generated by BKO Fusion. This inference could only be generated when the two fragments were fused together.

Besides handling contradictions, this example displays another strength of BKO theory. Consider the inference in Fig 13:

Here we start with Sciatic Neuropathy, and through a string of “is a” relations, we end at Inflammatory Disease. What makes this inference special is that it cannot be found in either MONDO or DO. Only by combining them can we draw the connection between sciatic neuropathy and inflammatory disease. Although sciatic neuropathy is not always described as an inflammatory disease, literature shows both that sciatic neuropathy is described as a disease or damage to the sciatic nerve [59] and that sciatic nerve injury triggers an inflammatory response [60]. Such insights are made possible by BKO fusion.

## 8 Conclusion

We presented a theory of representing and fusing probabilistic ontologies. This theory synthesizes the semantic expressivity and reasoning capabilities of both ontologies and BKBs without sacrificing the features, flexibility, or granularity of either. This theory depends on three key insights: (1) that disjoint classes can be mapped to a discrete random variable, (2) that generalizing DL reasoning principles to their probabilistic analogs naturally facilitates formal propagation of inheritance of probabilistic knowledge, and (3) that BKB theory and DL are matched in expressive granularity, enabling a natural synthesis founded on insights (1) and (2). Current methods for ontology merging require the resulting merged ontology to be consistent. Checking for and correcting inconsistencies is a costly process and may result in the rejection of true and useful information. BKO fusion overcomes this limitation by leveraging a BKB’s reasoning capabilities. As a result, all knowledge from the input ontologies will be included in the final fused one and reasoning can occur despite conflicting information. Additionally, the fused ontology will contain emergent information not present in the input ontologies individually, a powerful feature that means the fused BKO contains more knowledge than the union of its inputs.

Having completed the fundamentals of the theory, along with an outline of the reasoning process, our next steps will focus on deepening the theory. One track involves the information gained from fusing ontologies. Using BKO fusion, any practical number of ontologies can be fused together. However, at some point little information will be added when many ontologies with overlapping domains are fused. We will describe a method to quantify how much information is being added for each additional ontology. We will also focus on ontology alignment and its application to BKO fusion. One current limitation to our approach is its dependence on the availability of accurate ontology mappings. Recently work has been done focusing on automatically generated bridge ontologies, which would be well suited for our probabilistic framework and could be used to overcome the lack of a mapping between the ontologies used in fusion.
